# Patients Managing Their Medical Data in Personal Electronic Health Records: Scoping Review

**DOI:** 10.2196/37783

**Published:** 2022-12-27

**Authors:** Debby J Damen, Guus G Schoonman, Barbara Maat, Mirela Habibović, Emiel Krahmer, Steffen Pauws

**Affiliations:** 1 Department of Communication and Cognition Tilburg School of Humanities and Digital Sciences Tilburg University Tilburg Netherlands; 2 Department of Neurology Elisabeth-TweeSteden Hospital Tilburg Netherlands; 3 Department of Pharmacy Elisabeth-TweeSteden Hospital Tilburg Netherlands; 4 Department of Medical and Clinical Psychology Tilburg School of Social and Behavioral Sciences Tilburg University Tilburg Netherlands; 5 Department of Remote Patient Management & Connected Care Philips Research Eindhoven Netherlands

**Keywords:** patient-generated data, patient portal, personal electronic health record, patient activation, patient engagement

## Abstract

**Background:**

Personal electronic health records (PEHRs) allow patients to view, generate, and manage their personal and medical data that are relevant across illness episodes, such as their medications, allergies, immunizations, and their medical, social, and family health history. Thus, patients can actively participate in the management of their health care by ensuring that their health care providers have an updated and accurate overview of the patients’ medical records. However, the uptake of PEHRs remains low, especially in terms of patients entering and managing their personal and medical data in their PEHR.

**Objective:**

This scoping review aimed to explore the barriers and facilitators that patients face when deciding to review, enter, update, or modify their personal and medical data in their PEHR. This review also explores the extent to which patient-generated and -managed data affect the quality and safety of care, patient engagement, patient satisfaction, and patients’ health and health care services.

**Methods:**

We searched the MEDLINE, Embase, CINAHL, PsycINFO, Cochrane Library, Web of Science, and Google Scholar web-based databases, as well as reference lists of all primary and review articles using a predefined search query.

**Results:**

Of the 182 eligible papers, 37 (20%) provided sufficient information about patients’ data management activities. The results showed that patients tend to use their PEHRs passively rather than actively. Patients refrain from generating and managing their medical data in a PEHR, especially when these data are complex and sensitive. The reasons for patients’ passive data management behavior were related to their concerns about the validity, applicability, and confidentiality of patient-generated data. Our synthesis also showed that patient-generated and -managed health data ensures that the medical record is complete and up to date and is positively associated with patient engagement and patient satisfaction.

**Conclusions:**

The findings of this study suggest recommendations for implementing design features within the PEHR and the construal of a dedicated policy to inform both clinical staff and patients about the added value of patient-generated data. Moreover, clinicians should be involved as important ambassadors in informing, reminding, and encouraging patients to manage the data in their PEHR.

## Introduction

### Background

The beginning of most outpatient consultations is characterized by physicians going over the personal and medical information that is recorded in their patients’ personal electronic health records (PEHRs). This includes information about their patients’ current health problems and information about their vital signs, medication use, or known allergies. An up-to-date and accurate overview of this personal and medical information gives physicians a better sense of who is sitting in front of them and allows them to make appropriate and safe treatment-related decisions that correspond to their patients’ needs. In most cases, clinicians are responsible for updating their patients’ personal and medical data at the start of each consultation. However, this task can take up to 40% of the physicians’ time, which would rather be spent on direct patient care [1,2]. Instead of only physicians managing their patients’ personal and medical data (*core medical data*), patients can also play a role by entering, reviewing, and updating this information in their PEHR before or after each outpatient visit by themselves. Research shows that this active patient engagement is associated with various beneficial health-related outcomes, such as an increase in patients’ self-care and medication adherence, improved patient-physician relations, shared decision-making, and even improved clinical outcomes for patients with chronic illnesses [3-5]. It is for this reason that health care services strive to engage patients in the self-entry and self-management of their health care data by using technology such as patients’ PEHRs [6].

Over the past decade, identifying what determines whether patients are likely to engage with their PEHRs and how their engagement affects their clinical care has been a frequent topic of discussion [7-14]. The consensus is that less than half of the user population adopts a PEHR, and even less than one-third of the users actually use their PEHR records and manage their personal and medical data, with patients’ data management declining as age increases, lower digital skills, and being unable to fully understand and use health information in treatment-related decisions [15-18]. Studies have also shown that patients are less likely to self-manage their medical data when they find it difficult or unpleasant to use the data management tools [11,19-23] or when the practice is not endorsed by their health care providers [21,24].

**Figure 1:**
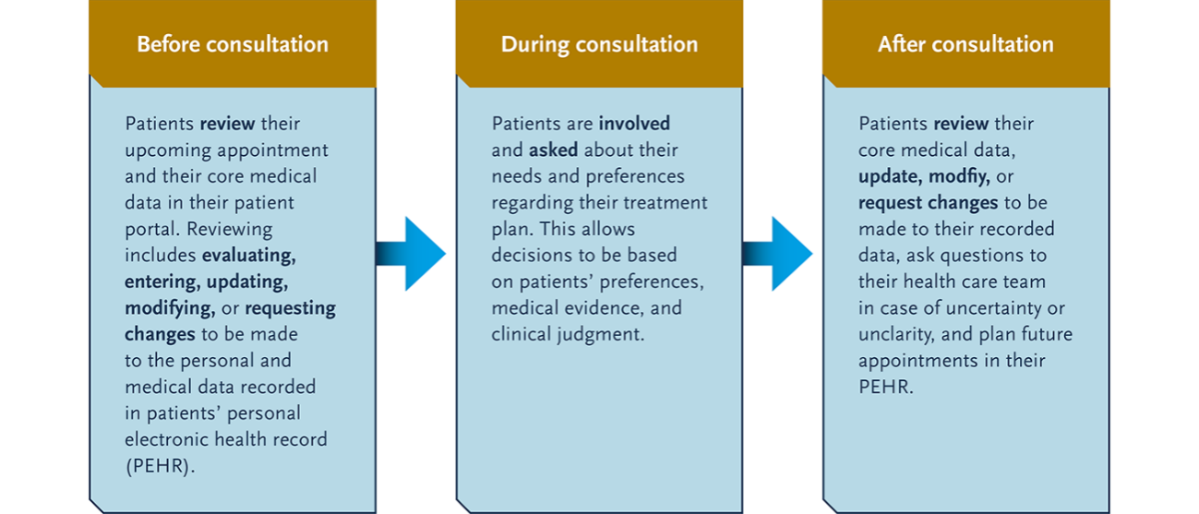
Active patient engagement in terms of patients generating and managing their personal and medical data throughout their care journey. This figure was partially replicated and adapted from Carman et al [30]. PEHR: personal electronic health record.

Although previous syntheses of the literature have been valuable in identifying the scope and potential causes of patients’ disengagement [7-10,13,14,25], they have some limitations. First, the most recent review [10] synthesized knowledge from studies published till 2018 and retrieved them from a very limited set of 3 databases. Second, previous reviews have focused only on consumers’ perceptions [7,10,13], patients aged ≥50 years [14], randomized controlled trials [8], or English publications [7,9,10,14], without providing an all-encompassing view on the patient-, care-, and system-related factors that drive or prevent patients’ data management. Most importantly, previous literature refrains from providing sufficient information about patients’ actual levels of engagement with their core medical data in their PEHR. The facilitators of and barriers to patients’ personal data management have previously been considered in relation to patients’ (future) portal adoption or access [25-27] or by basing patients’ level of engagement on log-in frequencies or the number of times they view a certain page in their PEHR [7-10,12-14]. In these cases, we do not know the extent to which patients who access their PEHR feel coresponsible or “empowered” [28] to actually use their PEHR in a meaningful way. We define meaningful use as patients actively sharing, reviewing, updating, or modifying their personal and medical data in their PEHR throughout their entire care journey (Figure 1). Our definition does not include patients who only access their portal and passively view the recorded information, but it does include patients who evaluate the information recorded in their PEHR. Certainly, patients are meaningfully using their PEHR when they closely examine (evaluate) their core medical data and decide to leave the information as it is, because they believe it to be correct and complete (Figure 1). However, we know that PEHRs often lack sufficient or up-to-date core medical information [29]. Therefore, in this review, our aim is to synthesize the existing literature by focusing on instances in which patients take actual action to provide or update their core medical data in their PEHR. This focus on data generation (sharing) and management (updating and modifying) allows us (1) to determine what drives patients toward or prevents patients from maintaining an up-to-date record and (2) to examine the associated impact that this active data management has on patients’ health and health care–related services.

To identify what may drive patients toward or prevent patients from taking on an active rather than a passive role when it comes to the management of their core medical data, we need to identify not only the type of data management activities patients perform within their portal but also the type of data that patients manage and how frequently they do so. Patients can engage differently with their PEHR depending on the personal and medical data they wish to share or update. Patients may be less inclined to share or update information about error-prone and sensitive data elements than to share or update personal and medical data that they are more confident or knowledgeable about. To date, it remains unknown whether the type of core medical information affects patients’ personal data management.

### Objectives

In this scoping review, we aimed to address the limitations of previous syntheses by exploring the barriers and facilitators that patients face when they decide to actively review, enter, update, or modify their core medical data in their PEHR throughout their care journey (Figure 1). We aimed to (1) identify the extent to which patients feel motivated or coresponsible for sharing, updating, and modifying their core medical data in their PEHR, and (2) examine the extent to which this engagement with a PEHR impacts the quality and safety of care and patients’ satisfaction with the care delivered. Answers to these questions will result in clear recommendations on how to maximally stimulate active patient involvement with PEHRs.

## Methods

### Search Strategy and Eligibility Criteria

This scoping review was conducted and reported in accordance with the PRISMA-ScR (Preferred Reporting Items for Systematic Reviews and Meta-Analyses Extension for Scoping Reviews [31]; Multimedia Appendix 1). The search protocol was preregistered with the Open Science Framework [32]. In April 2020, the MEDLINE (PubMed), Embase, CINAHL, PsycINFO, Cochrane Library, Web of Science, and Google Scholar web-based databases were searched to retrieve studies concerning patients’ management of their core medical data in an electronic patient portal. In March 2022, the MEDLINE database was re-searched to retrieve records that were published between April 2020 and March 2022. The reference lists of all primary and review articles were hand searched. Literature reviews were excluded, but practice briefs, fact sheets, white papers, and peer-reviewed publications (including conference proceedings) that focused on any type of population or study design (eg, qualitative, quantitative, or mixed methods studies) were included. The databases were searched for English or Dutch articles published between January 2000 and February 2020. We chose January 2000 as the starting point of the search because the 3 known early adopters of a web-based patient portal, the Palo Alto Medical Foundation (“MyChart”), the Beth Israel Deaconess Medical Center (“PatientSite”), and the Boston Children’s Hospital (“Indivo”), implemented their patient portals between the end of 1999 and the beginning of 2000 [33]. Our search strategy was developed in collaboration with an experienced research librarian (Multimedia Appendix 2) and targeted words related to electronic health records (eg, *patient portal* and *electronic health record*) combined with Medical Subject Headings terms related to patient engagement (eg, *patient participation*, *patient education*, *patient involvement*, and *patient engagement*) and the type of data being managed (eg, *medication reconciliation*, *medication verification*, *allergies*, and *intoxications*). To be included in the review, papers needed to focus on patients who actively handled their personal and medical data in a web-based patient portal (ie, entering, updating, or modifying; Figure 1) and identify either patient-, care-, or system-related determinants that influence this active patient involvement, or focus on the (perceived or examined) benefits or costs related to active patient involvement with a PEHR. Articles were excluded when they only included patients’ management of their core medical data in a PEHR as a secondary concept. Table 1 provides an overview of the checklist for full articles.

**Table 1:** Selection checklist for full articles.

Item		Inclusion
**Report characteristics**		
	Type of publication	Practice briefs, fact sheets, white papers, and peer-reviewed publications and conference proceedings. Exclude when the articles are systematic or scoping reviews; meta-analyses
	Date of publication	Between 2000 and February 2020; MEDLINE: re-searched in March 2022
**Study details**		
	Type of study or intervention	All types of studies are allowed to be included in this review (eg, randomized controlled trial, nonrandomized controlled trial, evaluation/usability, experimental, cohort/longitudinal, developmental, and pre-post design)
	Type of health data being managed	Core medical data being managed in a personal electronic health record (eg, medication regimen, vaccinations, allergies, medical and family history, and intoxications)
	Population	Both patients and clinicians

### Screening Rounds and Data Extraction

**Figure 2:**
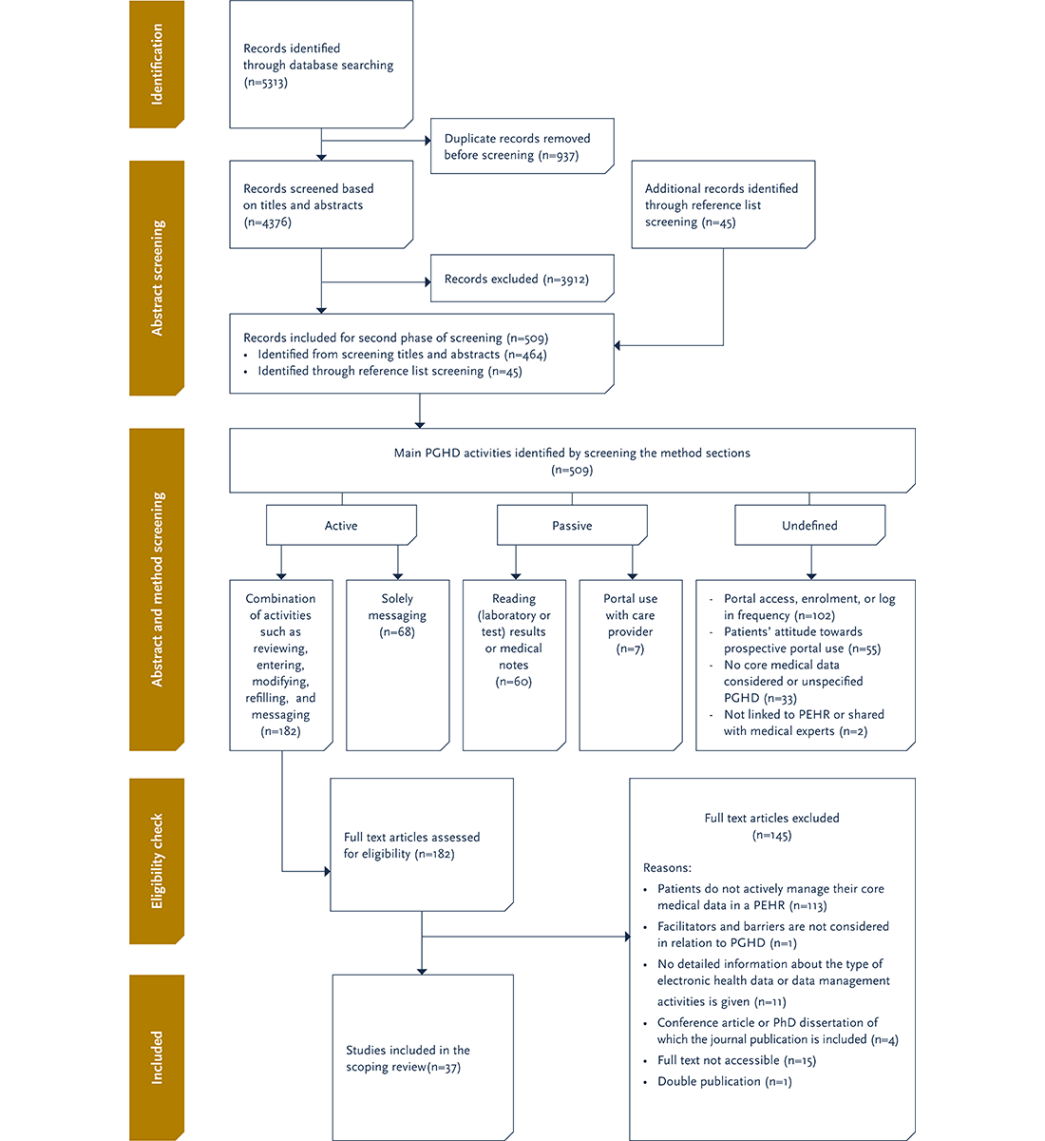
Flowchart for the identification, screening, and inclusion of articles in this scoping review. PGHD: patient-generated health data; PEHR: personal electronic health record.

The flowchart for the inclusion of articles in the scoping review is presented in Figure 2. The eligibility screening and data extraction form is presented in Multimedia Appendix 3. Searching the databases resulted in 5313 records that were imported into the reference manager, Mendeley (Elsevier). After duplicates were removed, 4376 (5313/4376, 82%) unique records were retained. The first author (DJD) used Mendeley to screen the identified records based on their titles and abstracts. A total of 45 (1%) additional records were identified through the screening of reference lists. This initial screening resulted in 509 records that were identified to be eligible for the review. However, after this initial screening, it remained unclear what kinds of activities patients performed within the PEHRs. Therefore, we diverged from our preregistered review protocol by administering an additional screening round. In this round, the first author (DJD) screened the Methods section of the 509 records to identify what kind of patient-generated medical data activities were included. This screening method identified 7 activities (Figure 2): active (ie, generating data, refilling, and messaging), passive (ie, viewing and portal use with health care provider), and undefined data management activities (ie, prospective use, portal access, log-in frequency, and portal enrollment). The first author (DJD) categorized the records into these 7 categories, and the second author (GGS) screened and reviewed a subset (51/509, 10%) of these records. Both authors discussed the screening method and the categorized subset until a consensus was reached. After the screening of the Method sections, 182 articles were found to be eligible for full-article screening. The full texts of these 182 records were subsequently screened by 4 authors (DJD, GGS, BM, and SP) in equally divided subsets. This resulted in 37 (20%) records that met the criteria for inclusion in this scoping review. The first (DJD) and second (GGS) authors then rated a subset of a mix of inclusions and exclusions, but no problematic cases were identified. The first author (DJD) then commenced with extracting the data from the 37 (20%) records according to the data extraction form (Multimedia Appendix 3).

## Results

### Description of the Included Studies

The general characteristics of the 37 included records are presented in Table 2. We rejected articles that only addressed patients who passively reviewed their data without making actual changes to their records (eg, the studies by Apter et al [34] and Jhamb et al [35]). We categorized the included studies as reporting on one or more of the following three categories (Table 3) [33,36]: (1) information about patients’ portal use, including the frequency of patients entering, updating, or modifying their core medical data; (2) patient and provider (perceived) facilitators of and barriers to the activities described in the first category, including usability, prototyping, and pilot studies in which portal features or tools were tested with specific end users; and (3) the impact of patients’ active involvement in the management of their data on patient care, including studies that focused on the quality of the data entered and the (perceived or examined) effects of patient-generated or patient-managed data on the quality, safety, cost-effectiveness, and patient or health care provider satisfaction of health care services. In further sections, we will report the findings of the included studies based on these categories.

**Table 2:** Study characteristics of the records included in the scoping review.

Number	Study	Country	Study aim	Sample	Type of data	Data activity	Portal	Data entry tools
1	Ali et al [37], 2018	United States	Evaluating the usability of a portal	Patients or caretakers of patients (n=23) with chronic conditions (diabetes, cancer, ulcerative colitis, or thalassemia)	Medical history	Reviewing and entering data	myNYP	None
2	Ancker et al [38], 2019	United States	Describing portal adoption rates and characteristics of patients who enter health data and their association with clinical outcomes	Patients with diabetes (n=53), of which 23 were pregnant and 30 were nonpregnant, and their physicians in obstetrics-gynecology (n=12) or internal medicine (n=4)	Blood glucose values	Entering data	Weill Cornell Connect (EpiCare)	None
3	Arsoniadis et al [39], 2015	United States	Evaluating the quality of patient-generated health data with a health history tool accessible via the web or a tablet	Patients (n=146) with an appointment at a surgery clinic, of whom 50 completed the intervention	Medical history, surgical history, and social history (including questions related to tobacco use, alcohol consumption, illicit substance use, and sexual history)	Entering data	EpiCare	Questionnaires
4	Bajracharya et al [40], 2019	United States	Evaluation of the family history module implemented in a patient portal and patients’ adoption of and experiences with the module	Patients (n=4223)	Family health history	Reviewing and entering and modifying data	PatientSite (electronic medical record of the Beth Israel Deaconess Medical Center)	Questionnaires
5	Bryce et al [41], 2008	United States	Exploring the usability of patient portal features and users’ intentions to pay fees for portal use for a diabetes management portal	Patients (n=39) with diabetes, with 21 patients allocated to the preportal group and 18 to the portal users group	Vital signs (blood glucose values)	Entering data	HealthTrak	Calculator
6	Chrischilles et al [42], 2014	United States	Exploring how patient-generated health data affects medication use safety among older adults	Nonclinical population (n=1075) with variety in medical backgrounds; most participants were experiencing stomach-related problems; 802 participants were allocated to use a patient portal, and 273 were allocated to a control group	List of allergies, medication list, problem list, and medical history	Entering data	Iowa PHR^a^ (stand-alone patient portal)	None
7	Cohn et al [43], 2010	United States	Evaluating the usability and analytic validity of the Health Heritage tool that helps patients to collect their family health history	Mixture of nonclinical and clinical participants (n=109), of which 54 were allocated to the intervention arm (Health Heritage) and 55 to the usual care arm	Family health history	Entering data	Health Heritage (stand-alone tool)	None
8	Polubriaginof and Pastore [29], 2016	United States	Comparing the accuracy and completeness of a tablet-administered problem list questionnaire to a problem list that was self-reported by patients	Patients with variety in medical backgrounds (n=1472); details were given for patients with hypercholesterolemia and diabetes	Problem list, medical history, family health history, and risk factors	Entering data	LMR^b^	Tablet questionnaire administered via the Hughes RiskApps life cycle cost software
9	Dullabhet et al [44], 2014	United States	Exploring how patients can be engaged to provide feedback on electronic health record content and how this feedback affects the accuracy of medical records	Patients (n=457) with chronic conditions (obstructive pulmonary disease, asthma, hypertension, diabetes, or heart failure); the number of providers and pharmacists interviewed is not provided	Medication list	Reviewing and modifying data	MyGeisinger (Geisinger Health System)	Web-based feedback forms
10	Eschler et al [45], 2016	United States	Exploring the usability of a patient portal, whether and how it helps patients to remember important health tasks, and whether it enhances patient engagement and agency in managing a chronic illness	Patients with diabetes and parents managing asthma for child dependents (n=19)	Immunization record	Reviewing and entering data	Three paper prototypes that represented features of a regional health cooperative portal’s interface were used	None
11	Hanauer et al [46], 2014	United States	Exploring the frequency, type, reasons, and outcomes of patient-initiated amendment requests	Patients (n=181) for whom amendment requests were made to various clinical departments and divisions but whose medical conditions were unspecified	Medical history, social history, intoxications, family health history, clinic notes, discharge summaries, and emergency department notes	Reviewing and modifying data	MyChart (Epic)	To initiate a chart amendment request, the patient had to contact the information management department by phone, by mail, fax or in person and obtain an amendment request form
12	Heyworth et al [47], 2013	United States	Testing a medication reconciliation tool to improve medication safety among patients who were recently discharged from the hospital	Patients (n=25) with chronic conditions (eg, diabetes, hypertension, prior myocardial infarction or stroke, hyperlipidemia, and heart disease)	Medication list	Reviewing and entering and modifying data	My HealtheVet (The Veterans Health Administration)	Secure Messaging for Medication Reconciliation Tool within the portal
13	Hill et al [48], 2018	United States	Exploring health care providers perceived advantages and disadvantages of PHR portal use	Health care providers (n=26) who treat patients with spinal cord injuries and disorders	Vital signs (blood pressure, pulse rate, and weight), medical history, immunization record, and medication list	Reviewing and entering data	My HealtheVet (The Veterans Health Administration)	None
14	Laranjo et al [49], 2017	Portugal	Examining portal use, associated patient demographics, and clinical variables	Patients (n=109,619), of whom 18,504 were portal users	Vital signs (height, weight, blood pressure, glycemia, cholesterol, and triglycerides levels) and allergies	Entering data	Tethered PHR provided by the National Health Service	None
15	Lemke et al [50], 2020	United States	Exploring primary care physicians’ experiences with the Genetic and Wellness Assessment tool for capturing patients’ family health history	Health care providers (n=24) who specialized in internal medicine, family medicine, or obstetrics/gynecology	Family health history	Entering data	Epic	Genetic and Wellness Assessment tool
16	Lesselroth et al [51], 2009	United States	Exploring the extent to which kiosk technology improves the reporting of patients’ medication history	Patients (n=17,868) visiting a chemotherapy facility	Medication list and list of allergies	Reviewing and entering and modifying data	See Data Entry Tools	Automated Patient History Intake Device accessed via computer terminal kiosk in the clinical waiting room
17	Murray et al [52], 2013	United States	To examine the capacity of 3 different electronic tools for collecting patients’ family health history	Patients (n=959) scheduled for an annual examination visit, of which 663 were allocated to the intervention arms (interactive voice response technology, patient portal, and waiting room laptop computer)	Family health history	Reviewing and entering data	Patient Gateway, LMR	The Surgeon General: My Family Health Portrait
18	Nagykaldi et al [53], 2012	United States	Examining the behavior and experiences of patients and primary care clinicians with regard to the Wellness Portal	Patients in primary care (n=560) who were in the randomized controlled trial; 3 clinicians, 2 office staff, and 6 patients in the pilot testing of the portal	Vital signs (weight), preventive services (mammography, diabetes education, and smoking counseling), wellness plan, symptom diary, medical history, medication list, problem list, list of allergies, and immunization record	Reviewing and entering data	Wellness Portal linked to the Preventive Services Reminder System	None
19	Nazi et al [54], 2013	United States	Exploring Veterans’ perspectives on receiving access to their personal medical information, which of its data elements they find most valuable, and how it affects their satisfaction, self-management, communication, and health care quality	Military service Veterans in the United States (n=688)	Medication list, list of allergies, and vital signs (eg, blood pressure, blood sugar, and cholesterol)	Entering data	MyHealth*e*Vet and Veterans Information System Technology Architecture	None
20	Park et al [55], 2018	Korea	Evaluating how and which users are generating and managing their personal and medical data	Patients with diabetes (n=16,729) and general users of the app (n=1536)	Vital signs (blood pressure, blood glucose levels, and weight); the functions list of allergies, medical history, and medication list were excluded because the number of users was relatively small (n=116)	Entering data	Mobile PHR known as My Chart in My Hand	None
21	Powell and Deroche [56], 2020	United States	Exploring the determinants of portal use among patients with multiple chronic conditions	Patients with multiple morbidities (n=500) with diabetes, heart failure, hypertension, and coronary artery disease	Vital signs (eg, weight and blood pressure)	Entering data	FollowMyHealth (AllScripts)	None
22	Prey et al [57], 2018	United States	Exploring the extent to which an electronic home medication review tool engaged patients in the medication reconciliation process and how this affected medication safety during hospitalization	Patients (n=65) arriving at the emergency department and their health care providers (n=20)	Medication list	Reviewing and entering and modifying data	AllScripts	Internally developed home medication review tool
23	Raghu et al [58], 2015	United States	Exploring the extent to which secure messaging helps patients to update their medication list in an ambulatory care setting	Patients (n=18,702) of a clinical practice that focused on surgical care for adults, of which 7818 had portal access	Medication list	Reviewing and entering data	Not specified	A secure messaging feature (alongside phone calls) was used by patients to update their medication list
24	Schnipper et al [59], 2012	United States	Investigating the extent to which a PHR-linked medications review module affects medication accuracy and safety	Patients in primary care (n=541), of which 267 were in the intervention arm	Intervention arm: medication list, list of allergies, and diabetes management information; control arm: family health history	Reviewing and modifying data	Patient Gateway, LMR	Patient Gateway medications module; electronic journals
25	Seeber et al [60], 2017	Germany	Validating the accuracy of VaccApp in helping parents to report their children’s vaccine history	Parents (n=456) of infants and children with suspected vaccine-preventable diseases (eg, influenza-like illness or infections of the central nervous system)	Immunization record	Reviewing and entering data	Vaccination app (VaccApp)	None
26	Sun et al [61], 2019	United States	Exploring how patients with type 2 diabetes use their patient portals and what determines their portal use	Parents (n=456) of children with diabetes, of which 178 used the app	Medication list, list of allergies, and medical history	Reviewing and entering data	Epic	Questionnaire for recording medical history
27	Tsai et al [62], 2019	United States	Exploring the characteristics of portal users and the activities that users perform within their patient portals	Patients (n=505,503), of which 109,200 were registered for a portal	Problem list, medication list, and list of allergies	Reviewing and entering and modifying data	MyChart (Epic)	None
28	Wald et al [63], 2010	United States	Exploring patients’ and health care providers’ experiences of using previsit electronic journals to record core medical data and survey data	Patients in primary care (n=2027 in the intervention arm and n=2345 in the postintervention survey) and 84 physicians	Arm 1: medication list, list of allergies, and diabetes items; arm 2: health maintenance, personal history, and family health history	Reviewing and entering and modifying data	Patient Gateway, LMR	Previsit electronic journals with tailored and untailored questions
29	Yu et al [64], 2015	United States	Exploring and identifying the needs and preferences of individuals with dexterity impairments when they use iMHere.	Patients with dexterity impairments (n=9)	Medication list and problem list	Entering reasons for taking medication and modifying medication reminders	Interactive mobile health and rehabilitation apps. iMHere is a system that connects smartphone apps to clinicians’ web-based portal.	MyMeds app (medication management) and SkinCare app (monitoring and reporting skin breakdown)
30	Zettel-Watson and Tsukerman [65], 2016	United States	Exploring the use patterns among users of web-based health management tools and identifying barriers to use among nonusers	Nonclinical population (n=166)	Vital Signs (cholesterol, blood pressure, and glucose levels; uploading data from a monitoring device)	Reviewing and entering data	Most participants used tools provided by their physician’s office, hospital, or insurance company (type of records unspecified)	None
31	Siek et al [66], 2011	United States	Testing the usability of an open source, web-based personal health app that provides older adults and their caregivers the ability to manage their personal health information during care transitions	Older adult patients with multiple morbidities (n=31)	Medication list	Reviewing and entering data	Colorado Care Tablet, personal health app	Pharmacy fulfillment and barcode scanning and a Prepare For Appointments wizard
32	Lober et al [67], 2006	United States	Exploring the barriers that older adults and disabled persons face when using PHRs	Nonclinical population (n=38) specified as low-income older adults with disabilities residing in a publicly subsidized housing project	Family health history, list of allergies, medication list, medical history, and immunization record	Reviewing and entering and modifying data	Personal Health In- formation Management System	A nurse was available to help with data entry
33	Arar et al [68], 2011	United States	To assess the facilitators of and barriers to Veterans’ use of the Surgeon General’s web-based tool to capture their family health history	Veterans (n=35)	Family health history	Entering data	My HealtheVet (The Veterans Health Administration)	The Surgeon General: My Family Health Portrait
34	Wu et al [69], 2014	United States	Assessing the content and quality of the MeTree family health history tool	Patients in primary care (n=1184)	Family health history	Entering data	MeTree	None
35	Cimino et al [70], 2002	United States	Exploring patients’ portal use, the cognitive effects of portal use and how it affects the patient–health care provider relationship	Patients (n=12) and health care providers (n=3)	Vital signs (height, weight, blood pressure, pulse, and temperature) and diabetes diary	Reviewing and entering data	Patient Clinical Information System, New York Presbyterian Hospital clinical data repository	None
36	Witry et al [71], 2010	United States	Exploring family practice physician and staff views on the (dis)advantages of PHR use	Health care providers (n=28) of a family medicine department	Medical history, medication list, and vital signs (blood pressure and glucose levels)	Entering data	Not specified	None
37	Kim and Johnson [72], 2004	United States	Exploring whether and how different types of data entry methods used by PHRs affect the accuracy of patient-generated data	Patients with disorders requiring treatment with thyroid hormone preparations (n=14)	Problem list and medication list	Reviewing and entering data	Password- protected website used to test data entry methods	Free-text entry (recall or abstraction) and selection methods

^a^PHR: patient health record.

^b^LMR: longitudinal medical record.

**Table 3:** Categorization of patient management papers and study type (N= 37).

Categories			Records^a^, n (%)		Study types and references
Frequency of portal use			27 (73)		Observational [38,42,49,55,56,58,62,70] Content analysis [44,46,51,63] RCT^b^ [42,53,59,63] RT^c^ [57] NRT^d^ [52] Cohort [43,61] Interview [44,47,50] Usability [47] Survey [54,65,70]
**Facilitators and barriers**					
	Patient-related	33 (89)		Observational [38,42,49,55,56,58,62,63,70] Content analysis [39,44,46,69] RCT [42,53,63] RT [57] Cohort [61] Interview [44,47,50,66,68,71] Usability [47,66,67] Prototype testing [45] Survey [40,54,65,68,70]	
	Provider-related	7 (19)		Content analysis [39,46,51] Interview [48,50,71] RCT [53]	
	System-related	28 (76)		Observational [55,63] Content analysis [44,46,51] NRT [72] RCT [42,53,63] Cohort [61] Interview [44,47,50,66,68,71] Prototype testing [45] Usability [37,41,48,64,66,67] Survey [40,54,65,68]	
Impact on patient care			26 (70)		Observational [29,38,42,63] RCT [42,53,59,63] NRT [52,72] RT [57] Cohort [43,60] Interview [44,47,48,50,68] Content analysis [39,44,46,51,69] Usability [47] Survey [40,54]

^a^The total number of records exceeds the total number of included studies because records contributed to more than one category.

^b^RCT: randomized controlled trial.

^c^RT: randomized trial.

^d^NRT: nonrandomized trial.

### Actual Use Information

#### Few Registered Users Enter Core Medical Data

**Figure 3:**
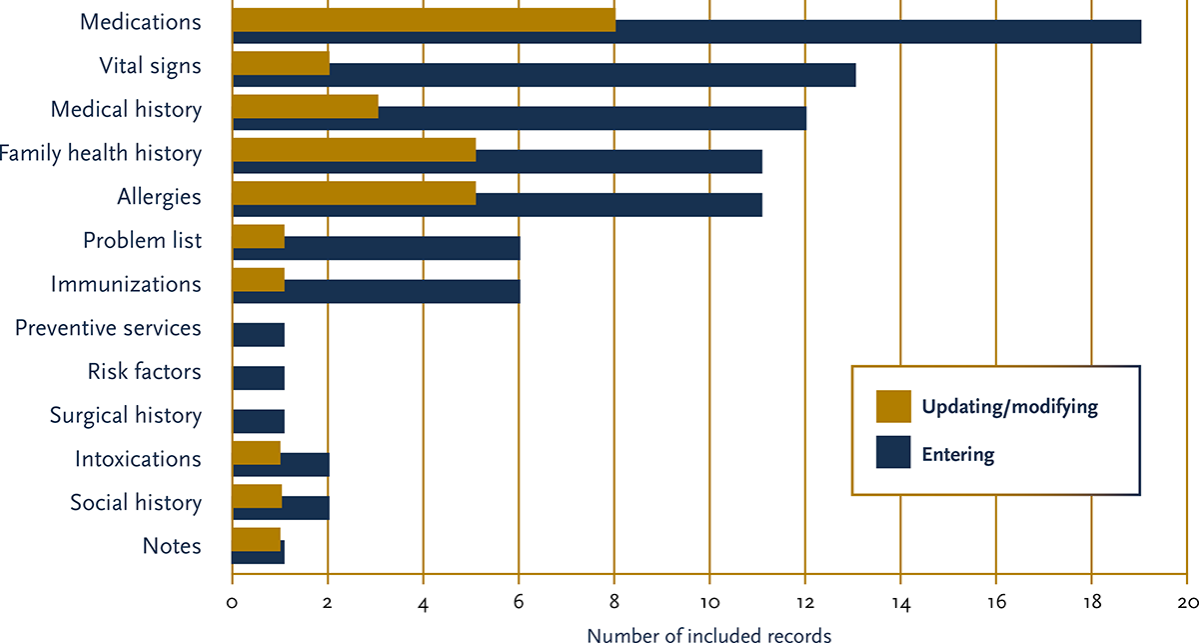
Distribution of the core medical data components managed (entered, updated, and modified) by patients. PEHR: personal electronic health record.

Figure 3 and Table 4 display the distribution of the core medical data components managed (entered, modified, or updated) by the patients in the included records. In more than half (25/37, 68%) of the included records, patients performed predefined data management tasks in which the usability of the tool or the effects of patients’ data management on data quality were explored, and 3 records explicitly reported that their patients wanted to update more information than they were allowed to [40,44,45]. Reviewing the 13 papers in which patients’ data management was not constrained by task demands [41,46,49,53-56,58,61,62,65,66,70] showed that the percentage of patients making changes to their core medical data ranged from 0.2% [46] to 22% [54] of registered users. Patients appreciated having insight into their recorded data but were otherwise not adding or updating this information [46,56]. A study investigating the number and content of amendment requests showed that over a period of 6 years, the number of patients requesting changes to their core medical data was extremely small relative to the number of patients requesting access to their patient records (0.2% of the access requests) [46]. Even when patients did request changes to their medical records (N=818), these changes were mostly related to clinical notes (308/818, 37.7%) and discharge summaries (84/818, 10.3%) [46] and not to the core medical data components (eg, admission history and physical; 19/818, 2.3%). In line with this, studies have shown that portal features that only allowed patients to view their medical information [54,61,62,70] or to message their health care provider [41,54,56] were more frequently used than features that allowed the self-entry of medical data. These passive features were valued more than self-entry features [41,54]. When patients did use self-entry features, they seemed to prefer to enter information about their vital signs (eg, blood pressure, blood glucose values, and weight) compared with other core medical data components [41,49,53,55,65,70].

**Table 4:** Distribution of core medical data components managed and associated tasks across the included records.

Data component and activity, constrained or unconstrained by task demands				Records, n (%)		References
**Generating core medical data (entering and sharing data)**						
	Constrained			24 (64.8)		[29,37-40,42-45,47,48,50-52,57,59,60,63,64,67-69,71,72]
	Unconstrained			13 (35.1)		[41,46,49,53-56,58,61,62,65,66,70]
	**Medications**					
		Constrained	12 (32.4)		[42,44,47,48,51,57,59,63,64,67,71,72]	
		Unconstrained	7 (18.9)		[53-55,58,61,62,66]	
	**Vital signs**					
		Constrained	5 (13.5)		[38,48,59,63,71]	
		Unconstrained	8 (21.6)		[41,49,53-56,65,70]	
	**Medical history (including personal history)**					
		Constrained	8 (21.6)		[29,37,39,42,48,63,67,71]	
		Unconstrained	4 (10.8)		[46,53,55,61]	
	**Family health history**					
		Constrained	10 (27)		[29,40,43,50,52,59,63,67-69]	
		Unconstrained	1 (2.7)		[46]	
	**Allergies**					
		Constrained	5 (13.5)		[42,51,59,63,67]	
		Unconstrained	6 (16.2)		[49,53-55,61,62]	
	**Problems list (including symptom diary and health conditions and issues)**					
		Constrained	4 (10.8)		[29,42,64,72]	
		Unconstrained	2 (5.4)		[53,62]	
	**Immunizations**					
		Constrained	5 (13.5)		[39,45,48,60,67]	
		Unconstrained	1 (2.7)		[53]	
	**Preventive services**					
		Constrained	0 (0)		—	
		Unconstrained	1 (2.7)		[53]	
	**Risk factors**					
		Constrained	1 (2.7)		[29]	
		Unconstrained	0 (0)		—	
	**Surgical history**					
		Constrained	1 (2.7)		[39]	
		Unconstrained	0 (0)		—	
	**Intoxications**					
		Constrained	1 (2.7)		[39]	
		Unconstrained	1 (2.7)		[46]	
	**Social history**					
		Constrained	1 (2.7)		[39]	
		Unconstrained	1 (2.7)		[46]	
	**Clinical notes, discharge summaries, and emergency department notes**					
		Constrained	0 (0)		—	
		Unconstrained	1 (2.7)		[46]	
**Managing core medical data (updating, modifying, and requesting changes to data)**						
	Constrained			8 (21.6)		[40,44,47,51,57,59,63,67]
	Unconstrained			2 (5.4)		[46,62]
	**Medications**					
		Constrained	7 (18.9)		[44,47,51,57,59,63,67]	
		Unconstrained	1 (2.7)		[62]	
	**Vital signs**					
		Constrained	2 (5.4)		[59,63]	
		Unconstrained	0 (0)		—	
	**Medical history (including personal history)**					
		Constrained	2 (5.4)		[63,67]	
		Unconstrained	1 (2.7)		[46]	
	**Family health history**					
		Constrained	4 (10.8)		[40,59,63,67]	
		Unconstrained	1 (2.7)		[46]	
	**Allergies**					
		Constrained	4 (10.8)		[51,59,63,67]	
		Unconstrained	1 (2.7)		[62]	
	**Problem list (including symptom diary and health conditions and issues)**					
		Constrained	0 (0)		—	
		Unconstrained	1 (2.7)		[62]	
	**Immunizations**					
		Constrained	1 (2.7)		[67]	
		Unconstrained	0 (0)		—	
	**Intoxication**					
		Constrained	0 (0)		—	
		Unconstrained	1 (2.7)		[46]	
	**Social history**					
		Constrained	0 (0)		—	
		Unconstrained	1 (2.7)		[46]	
	**Clinical notes, discharge summaries, and emergency department notes**					
		Constrained	0 (0)		—	
		Unconstrained	1 (2.7)		[46]	

#### Continued Use Drops as Time Increases

Of the 37 included studies, 23 (62%) provided information about the frequency of patients’ portal uptake [38-40,42-47,49-51,53-58,61-63,65,70]. Most of the sample (>50%) used the portal’s features [42,47,53,54,70] or specific tools [57], such as an app [43], electronic journal [63], or a computer terminal kiosk in the lobby [51], to enter or update their core medical data in only 9 (24%) of these records. In the remaining studies, a minority of patients (ranging from 0.04% to 44.16% of the population) used the portal’s features [45,46,49,55,56,58,61,62,65], an implemented flow sheet [38], a questionnaire [39], a feedback form [44], or a family health history module [50] to manage their core medical data. Most of these records identified patients’ use patterns at a specific time point, and only 19% (7/37) of the records explicitly considered patients’ frequency of portal use over time [42,49,53-55,61,70]. These latter studies showed that although active portal users usually have more multiple inputs than passive users [42,49], continued use is very limited. Users who manage their data for longer than a year represent only 5% to 9% of the user population [42,53-55,61], and continued use further decreases as time increases [45,55,61,70]. In the remainder of this paper, we explore what prevents patients from actively managing or helps patients to actively manage their core medical data.

### Factors Affecting Active Data Management

**Figure 4:**
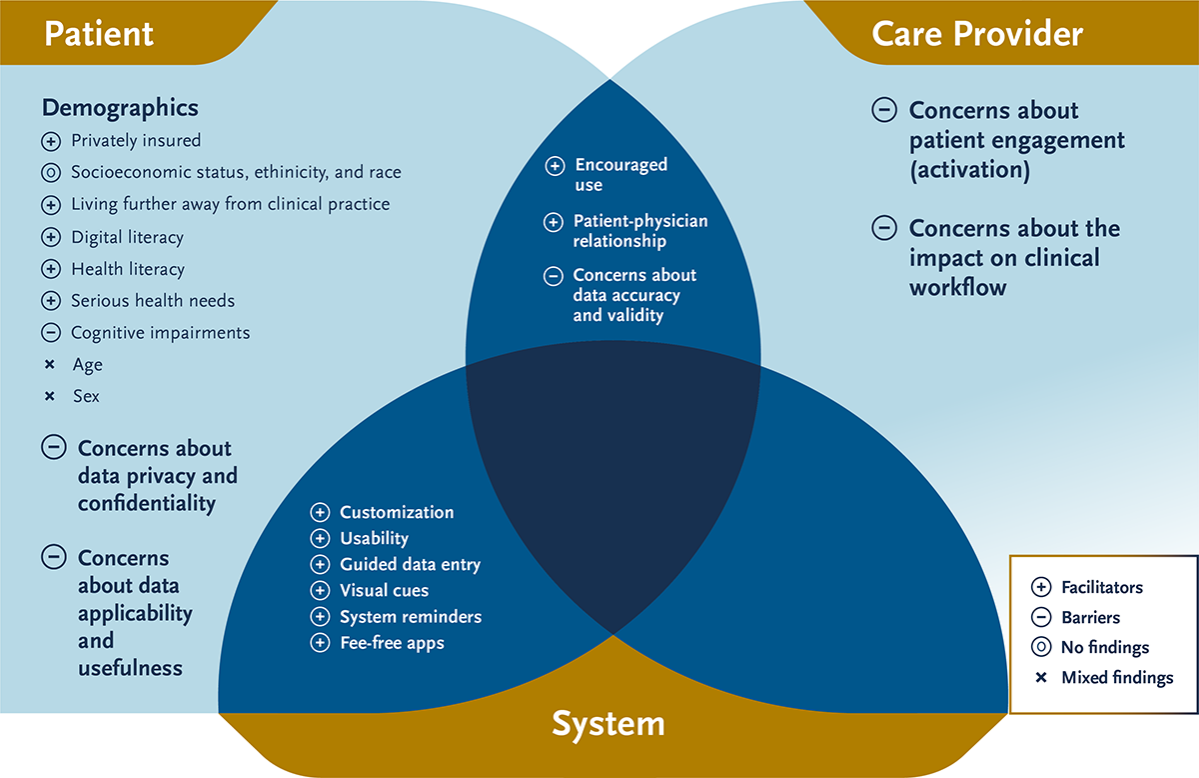
Patient-related, health care provider–related, and system-related factors affecting patients’ management of their personal and medical data.

We categorized the facilitators and barriers associated with patients actively managing their core medical data through a patient portal into one of the three categories: those dealing with patient characteristics, those dealing with health care provider characteristics, or those dealing with system characteristics. A brief overview of how the important factors affecting patients’ personal data management are related to each other is presented in Figure 4.

### Patient-Related Determinants

#### Overview

We identified the following 6 themes that determined whether patients entered, updated, or modified their core medical data: patient demographics; digital and health literacy; concerns related to the accuracy, validity, privacy**,** and confidentiality of recorded data; misconceptions about the applicability; and usefulness of patient-entered data.

#### Patient Demographics

There is little consensus on whether and how a patient’s age or sex influence active data management. While 6 retrospective studies indicated that younger patients are more likely to manage their core medical data [38,42,49,58,61,65], 4 similar studies showed the exact opposite pattern [55-57,62]. In all records, comparisons were predominantly made within rather than across age categories. Taken together over all included records, we see that the age of active portal users ranges from approximately 30 to 70 years [38,42,55,61,62,65], with the most active users being more likely to be in their 30s or 60s [62]. In terms of patients’ sex, in 4 retrospective studies, active portal users were more likely to be male than female [42,49,55,61], but 2 other similar studies showed the opposite [62,65]. Thus, age and sex are not very indicative of patients’ level of involvement in the generation and management of their core medical data. It may be more informative to look at other patient demographics.

A total of 5 (13.5%) retrospective studies showed that compared with inactive or less active users, active portal users are more likely to be privately insured [58], to have a higher median household income and education level [61], to live farther away from a clinical practice [56], or to reside in urban centers [49,61]. Furthermore, 3 retrospective use pattern studies did not find any significant differences in socioeconomic status, race, or ethnicity of active versus nonactive users [38,42,62]. In 2 other retrospective studies [42,57] and 1 cluster randomized controlled trial [53], active users were found to be digitally competent with a computer or tablet and were already using technology to improve their health [53]. In addition, 3 retrospective user evaluations showed that active users wanted to ensure that their provider had the most accurate and complete information [40] and reported to have already managed their medical data offline [42] or on the web [65]. We also found that active use might depend on patients’ medical condition and health needs, as user pattern studies have shown that active users have a more serious health condition [38,42,53,56,57,61,70] and more clinical encounters [38,62] than other users. In a related vein, a randomized pilot study showed that active users were more interested in improving their understanding of their medical problems and treatments [54]. A usability study showed that cognitive impairments (eg, Alzheimer disease and dementia) and physical limitations (eg, hearing and vision impairments and joint diseases) negatively affected patients’ ability to independently manage their medical data in an electronic system [67].

#### Digital and Health Literacy

Limited internet or computer access, digital illiteracy, and computer anxiety are barriers to patients entering and modifying their core medical data electronically [67,68]. Interviewed users of a web-based family health history tool reported that a lack of knowledge about how to use a computer or web-based technology might limit patients’ ability to manage their data electronically without assistance, especially when tasks become more complex [68]. In addition, older adult patients with disabilities reported that their lack of understanding or knowledge of the terminology used for core medical data and how they should report it prevented their data entry [67]. This negative impact of health literacy on active data management was also addressed by interviewed primary care physicians evaluating another implemented family health history tool [50] and by patients recording their family health history in a retrospective data analysis [69] and a user evaluation study [40].

#### Concerns About Data Accuracy and Validity

An interesting factor that might explain whether patients manage their core medical data is their belief and reassurance that they are not bypassing clinical staff by directly entering or modifying their data in their record [44,45,66]. Patients with multiple morbidities [66] and patients with diabetes or parents managing asthma for their children [45] reported that they preferred having health care providers updating their medical record on their behalf, in fear that their own modifications might alter their physicians’ information. In addition, interviewed patients with chronic conditions (ie, chronic obstructive pulmonary disease, asthma, hypertension, diabetes, or heart failure) who were reviewing and modifying their medication list indicated that they found it reassuring to know that all recommended changes were first checked by their provider before they were actually recorded in their medical records [44]. This reassurance can be corroborated by implementing visual features or cues into the interface that convey that patients are modifying personal information that is independent from their physician’s records [66]. Patients might also fear that they will provide inaccurate information to their caregivers because they cannot reliably recall medical information such as their family health history [40,43]. Patients who generated their family health history using prepopulated questionnaires stressed that they wanted to include this uncertainty in their records, explicitly stating that they would be more willing to share medical information if they could provide more contextual information to the reported data [40].

#### Concerns About Data Privacy and Confidentiality

Concerns about data loss and breach of privacy further prevent patients from maintaining their medical records electronically [40,65,68,71]. Patients seek the assurance of data confidentially and protection of their privacy. In a focus group interview, health care providers voiced that patients might fear that their identity might be stolen or that they might purposely omit medical information in fear that it might affect their health insurance or future employment [71]. This concern was indeed confirmed by patients evaluating an implemented family history module in a survey [40] and interview study [68] and by a nonclinical population reporting on their experience with web-based health management tools [65]. Owing to privacy and autonomy concerns, patients do not prefer to share identifiable information, such as their relatives’ names and ages [40].

#### Perceived Applicability and Usefulness

(Mis)conceptions about the applicability and usefulness of patient-generated health data may also prevent patients from taking on a more active role in the management of their personal and medical data via a PEHR. As was mentioned by interviewed patients [66] and interviewed health care providers [71], patients may not see the need to manage their medical information in a web-based portal, as they assume that their providers have access to and share more medical information among specialists than they actually do. Moreover, patients reported that not knowing the benefit of managing and updating medical information [65] or not knowing whether their health care provider actually used the information and found it to be useful [63] prevent their active participation.

### Health Care Provider–Related Determinants

#### Overview

Encouraged use by health care providers and the patient-clinician relationship are identified as the 2 important factors determining whether patients actively manage their core medical data. However, we noticed that health care professionals’ recommendations to use the system are dependent on whether they believe that there are benefits associated with patient-entered data in terms of data quality and reliability and cost-effectiveness.

#### Encouraged Use

Being encouraged by health care providers to manage core medical data plays an important role in the adoption and continued use of PEHRs among patients. First, in both a qualitative content analysis of patient-initiated amendment requests [46] and in a retrospective use pattern study by Ancker et al [38] in which patients managed their blood glucose values, it was suggested that the low amount of generated data was caused by patients not knowing whether they could make changes to their records or how they should go about it. Second, most (84%) respondents voiced that they used web-based health management tools because they were recommended to do so by their clinician [65]. Clinicians also realized that their own recommendations are important and that reminding patients to use the tools is an important activator of portal use [53]. Clinicians even went so far as to suggest that portal use could be a prerequisite for receiving regular care [53]. In addition, showing the added value of patient-generated health data during an outpatient visit might stimulate patient participation [45,65,67]. Patients with multiple morbidities in a retrospective user pattern study indicated they would stop using tools to record and maintain their core medical data if they did not have someone showing them how to use them, especially when they found it to be difficult to use the tools [65]. In particular, older patients with disabilities both seek and need assistance when it comes to entering and modifying their electronic core medical data [67].

We identified several beliefs that health care providers have about patient-generated and patient-managed medical data that may determine whether they are likely to encourage or assist their patients in managing their core medical data in their PEHR. First, health care providers are often unaware of the benefits that are associated with patients’ management of their own data [71]. Second, health care providers do not believe that their patients are motivated [71] or able to provide and maintain accurate and reliable information [44,48,71]. Moreover, health care providers may believe that reviewing patient-entered data may have a significant impact on time spent on outpatient visits and practice workflow [39,46,48,50]. Interviewed physicians who treated patients with spinal cord injuries and disorders voiced concerns that a patient’s medical and emotional state may affect their ability to record their data in a reliable fashion and that if patients misinterpret data retrieved from the portal, it might negatively affect their own documentation [48] or treatment information [71]. Pharmacists [44] and family physicians [71] were also skeptical about their patients’ ability to enter core medical data accurately. Physicians of a family medicine department explicitly voiced concerns that patient-entered data might be subjective and that health care providers should, therefore, always be in control of data input. Physicians stated that their patients may not know what is appropriate to put in their health records, causing them to enter information that is verified by a professional. They even believed that allowing their patients to enter information into their medical records might facilitate narcotics abuse because patients could inappropriately request or elicit prescriptions [71]. Furthermore, the time saved by having patients enter their own data may be counterbalanced by the time it takes for providers to review patient data [39,46]. Health care providers who treated patients with spinal cord injuries and disorders stressed that checking the patient portals impacts their time and workflow [48]. This view was shared by health care providers who specialized in internal (family) medicine, obstetrics, and gynecology in a study that explored their initial experiences with a family history screening tool implemented in a patient portal. Physicians reported a lack of time for using the tool and stressed that patient-generated and -managed data may only benefit their workflow if patients are able to fill out all the information before their outpatient visit [50].

#### Patient-Clinician Relationship

Patients testing a medication reconciliation tool via a secure messaging feature within the portal indicated that they appreciated the possibility of communicating directly with health care providers when they had questions about their medications or wanted to request refills. Most (90%) users said they would use the tool again, frequently emphasizing how it allowed them to have instant access to their health care provider [47]. On a related note, patients may refrain from managing their medical data if they want to avoid communicating with their clinicians. Patients with diabetes and parents managing asthma for dependent children voiced that they would rather not use the secure message feature when they did not trust or like their health care provider [45]. This study recommends design implications for the portal that could amplify the positive aspects of the patient–health care provider relationship, such as profile pictures accompanying health care providers’ messages or allowing patients to view or hide profiles from a care team in the portal.

### System-Related Determinants

#### Overview

Patients’ satisfaction with the system used to collect and maintain their core medical data is an important factor that stimulates active data management [44,64]. A total of 6 main themes emerged from the data extraction that concerned system-related facilitators and barriers affecting patients’ satisfaction with the tools used to record their medical core data: the level of customization, usability of the system or tool, guided versus free data entry, presence of visual cues, reminders, and fee-free access to the system/tool.

#### Customization

A total of 4 studies stressed the importance of offering a level of customization to patient portals [45,63,64,66]. To increase the usability of the system, patients could be allowed to prioritize frequently used portal features [45,63] by, for instance, adding these features to the front page of their portal [45]. Patients also prefer to personalize the system by assigning a personally selected background [64] or self-selected icons for portal features [66], increasing or decreasing the size of these buttons/icons [64], and changing the background and text colors to improve the readability of the portal [64].

#### Usability

Patients’ (continued) use of their electronic patient portal to generate and update their core data depends on the perceived complexity and thus the usability of the system or tools used [37,45,47,63,64,66,68]. Failure to record and maintain core medical data might result from patients not finding the area where it should be recorded [45] or because patients might misinterpret medical terms or encounter terms within the portal that they do not understand, causing frustration and self-doubt [37]. In general, participants prefer to have clear on-screen instructions and directions [53,64,66,68] and short drop-down menus [53]. Using thematic colors also improves the usability of a system [64]. Patients also prefer to have access to previously entered data and to be allowed to mark this information as unchanged when updating their core medical data in the system [63].

#### Guided Data Entry

Unless patients are being asked to enter information about simple diagnoses or prescriptions, systems should use guided entry of data elements [55,66,72]. Patients in 5% (2/37) of studies experienced problems during medication reconciliation when asked to enter their medication names into the system [55,66]. It was for this reason that they were reluctant to provide additional dosage and scheduling information [66]. Patients prefer a less textual way of adding medications to their list, voicing that free-text entry is too complex and time-consuming [66]. To aid the reviewing process, a prepopulated medication form [55] or a barcode scanning function [69] could be used, especially when patients need to report on a large number of medications [55]. Autofilling processes also give patients some reassurance about the accuracy of their data entry [66]. Free-text entries are undesirable when patients are asked to add information to their problem list, as they may be inclined to include extraneous information that does not contribute to the identification of a primary diagnosis [72]. However, in a study exploring patients’ experiences with a family history tool [40], patients reported on the danger of using closed answer options. The patients expressed concerns that some answers did not allow for sufficient granularity and reliability, arguing that their family history was often far more complex than what they were allowed to record. These patients also preferred to receive more clarity and information about the diseases that they were asked to report. Allowing patients to provide contextual information when they have the desire to do so might reassure them about their answers’ validity [40].

#### Visual Cues

Implementing visual feedback facilitates data entry by patients and patients’ satisfaction with using the system. For instance, providing medication pictures alongside a selected medication assists patients’ medication reconciliation [51] and allows them to confirm whether it is the correct medication to add [66]. In addition, patients prefer to receive clear feedback when performing an action within the system, such as seeing a medication being highlighted after they suggest it should be deleted from their list [66]. Visual feedback in the form of using red and green colors also helps patients to take further actions such as scheduling alerts to take the medication when a new medication is added to the list [64]. Using colors is also beneficial when they are used to demarcate separate body parts, helping patients to correctly specify the location of the problem skin areas [64].

#### Reminders

If reminded to do so, patients are more likely to use the portal before and after their outpatient visits [26]. Reminders generated through the portal stimulate patients to access their records [26] and enter information about their medications, allergies, and vital signs [54].

#### Fee-free Apps

Providing applications without charge [41] that can be downloaded by patients as well as by a more general group of users [55] stimulates the accumulation of patient-generated core medical data. A study that focused on patients’ diabetes management [41] showed that patients believed that implementing fees for portal access would significantly reduce their tendency to use the portal for the self-management of their diseases. The implementation of portal fees seemed unfair according to patients because health systems also benefit from patients’ self-management of their disease. Patients believed that introducing fees would increase inequities between patients who can and cannot afford using the portals, and they also feared that costs would increase when previously free services would start requiring payment [41].

### Impact on Patient Health and Health Care Services

**Figure 5:**
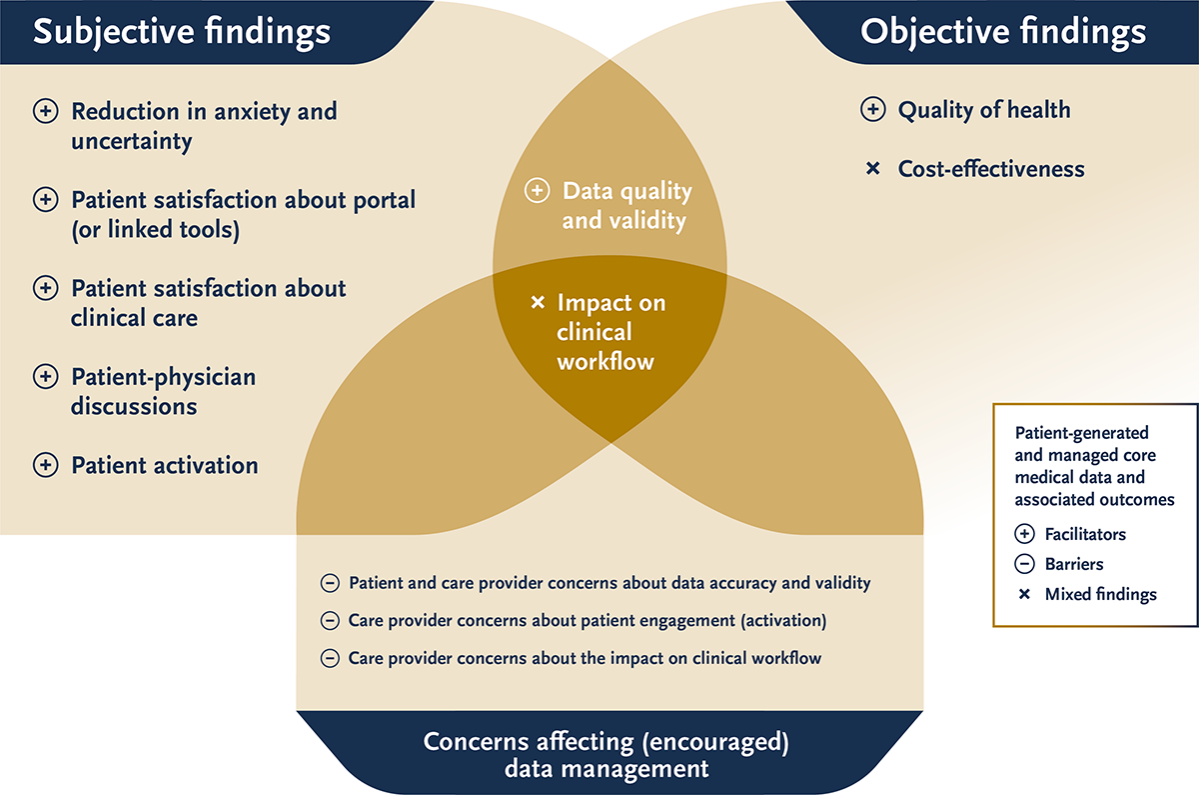
Impact of patient-generated health data (PGHD) on patients’ health and health care–related services and how this impact is associated with the important concerns regarding PGHD raised by patients and health care providers.

This section describes the impact of patients’ data management on the quality and safety of patient care, psychological outcomes for patients, patient engagement, patient satisfaction, and clinical workflow. Figure 5 presents the important subjective and objective outcomes identified and how they are related to the concerns of both patients and health care professionals.

#### Data Quality and Validity

Clinicians’ concerns about the quality and validity of patient-entered data seem to be unfounded. Observational [29], experimental [52,57,72], usability [47], cohort [60], and content analysis [44,46,69] studies have shown that medical records are completer and more accurate when the data are generated by patients themselves. Patients are able to accurately self-report on their diagnoses [29,72], medications [29,44,47,57], medical or surgical history [46], family health history [52,69], or their children’s vaccination history [60]. Patients request changes to their core medical data especially when this information is incomplete [46,47,59] or incorrect [46], and these requests are approved in approximately half [46] up to 80% [44] of cases. Studies have reported on improved medication reconciliation [44,47,51,57,59], arguing that patients’ management of their medical data makes them more attentive to medication safety and monitoring [42,44,47] and even helps clinicians to identify (potential) lethal medication discrepancies [51]. In addition, the quality and validity of patients’ problem lists [29], immunization records [60], and family health history [43,52,69] improves when patients enter and manage their own medical data. Clinicians even felt that the risks identified because of patients entering their family health history helped them to make informed changes to their patients’ medical management [50]. Pharmacists reported being surprised to learn about patients’ willingness and ability to report their medications accurately, even when patients were taking >20 medications or were taking medications that had been prescribed by physicians who were not part of the current health system [44]. Only 1 content analysis study did not show the added value of patient-generated data [39]. In this study, patients entered information about their medical, surgical, and social history, using closed question questionnaires with “yes” and “no” answer options. Patients were allowed to give additional information in the comments section. The researchers concluded that the new information added to a patient’s record often lacked sufficient granularity to be found meaningful. However, they did not reflect on how the closed nature of the questionnaire could have contributed to this outcome.

#### Quality of Health

Another theme we identified was a significant objective [38,53] and subjective [42,63] improvement in patients’ health because of them actively managing their medical data. First, an observational study of patients with diabetes who were uploading (and thus tracking) their blood glucose values showed a significant drop in their average BMI and mean glycated hemoglobin values compared with nonuploaders (nontrackers) [38]. Second, patients who entered and tracked their vital signs and preventive services were more likely to receive all recommended immunizations than control groups [53]. These objective findings are corroborated by patients’ self-reports [42,63]. Older adults reported more changes in medication use and improved medication reconciliation behaviors than less active recorders and nonrecorders. These patients also reported more side effects [42]. In a similar vein, patients in primary care who entered and modified their lists of medications and allergies felt that their health care provider had more accurate information about them and that this improved the quality of care at the visit [63].

#### Psychological Outcomes for Patients

Insight into medical data might reduce anxiety and uncertainty in patients. This point was explicitly raised by interviewed health care providers who were evaluating a tool that helped the patients under their care to report on their family health history to identify possible genetic diseases [50]. Patients felt less anxious when the tool identified no increased risk and they were able to discuss the findings with their clinician.

#### Patient Engagement

We identified two themes in this subsection: (1) the extent to which patients’ data management improves patient-physician discussions and (2) feelings of ownership among patients and future patient participation.

#### Improved Patient-Physician Discussions

Patients who update their core medical data before an outpatient visit, feel better informed [44] and better prepared for the visit [44,63,70] and experience improvement in their interaction with their health care providers [50-52,54,59,63,65,70]. Patients indicate that they can provide more comprehensive information about complex and sensitive health issues at home than in their physician’s office because in the latter case, they feel more stressed and uncomfortable [40]. Patients [43,52] and primary care physicians [50] believe that patients who update their family health history are more aware of its (medical) importance, facilitating both patient-physician [50,52] and patient-family [43,50] discussions about associated family history–related health risks and ways to improve their health. Patients who manage their vital signs data prepare their questions before visiting their provider [70], thereby improving treatment-related discussions and decisions [65,70]. Regarding medication reconciliation, nurse practitioners mentioned that allowing patients to review, update, and modify their medication lists improved their medication dispensing information and identification of errors [51,59]. In their turn, practitioners [51] and patients in primary care [59] stated that patients asked more questions about their regimens [51], were more likely to report adverse reactions [51] or to address medication-related problems and new symptoms [59], and requested more refills for medications that were nearing their expiration date [51]. Active patients feel more confident when asking questions about medications during their outpatient visits [44], and they recall more questions that they want their physicians to answer. Patients also feel that such preparation saves time during the visit [63] or even reduces the need for an outpatient visit [44]. This viewpoint is shared by primary care clinicians, who stress that they would recommend that other clinicians ask their patients to review, update, and modify their list of medications, allergies, and diabetes items before an outpatient visit [63].

#### Patient Activation

Patients who generate and manage their own medical data feel that they have more control over their health care and health-related decisions [40,44,53,65,70,71]. A randomized controlled trial comparing patients who managed their core medical data against nonactive patients showed that active patients were not only more confident and knowledgeable about their health in general and about making health-related decisions but were also more likely to actually take action to improve their health [53]. These findings are supported by studies that focus on patients who managed their family health history [40,68], vital signs [65,70,71], medical history [71], and medications [44,71]. Patients feel that their participation improves their clinician’s knowledge [40,70]. Patients experience a sense of ownership when they manage their own medical data [70] and report that they consider their contributions to be valuable to an extent that makes them feel empowered [40] and motivated [68] to improve their health condition. This viewpoint is shared by family physicians [71] and health care providers who treat patients with spinal cord injuries and disorders [48]. These clinicians feel that if patients maintain their medical data, they may become more organized and adherent to medications [48] and improve their involvement in their care, which may result in better outcomes [71].

#### Patient Satisfaction

Patients were generally satisfied with the tools that they used to update their medical data [43,63,64,68]. Only 2 records discussed whether active management of data by patients affected patients’ satisfaction with their clinical care [40,63]. One of these records measured patient satisfaction using a 1-item survey question [63], showing that 37.7% of the respondents were more satisfied with their visit after they had first entered or updated their medical information using electronic journals implemented in a patient portal. The second study found that their patients were more satisfied with reviewing their free-text responses after they had entered or updated their family health history in their web-based records [40]. In the comment section of that study [40], patients reported that they felt welcomed, cared for, and safe when asked to share their medical information.

### Impact on Clinical Workflow and Costs

A study that interviewed health care providers who treated patients with spinal cord injuries and disorders found that health care providers believed that patient-generated health data collected via patient portals can improve the coordination of medical care, especially for those patients who receive health care in nonclinical settings [48]. However, we found mixed evidence concerning the effects of patients’ active management of their medical data on clinical and patient throughput. Both clinicians [57,70] and patients [63,70] believed that asking patients to review and update their medical data before an outpatient visit positively affects clinical throughput because consultations can be executed more efficiently. For instance, pharmacists and physicians stated that they spent half of the usual amount of time on medication reconciliation on outpatient visits when patients generated this information themselves [44]. Active involvement of patients in the generation and management of their data may even reduce the need to schedule an outpatient visit [44], especially when physicians can address their patients’ questions via a secure messaging feature [48]. However, interviewed family physicians were concerned that patient-generated data would negatively impact consultation time if it required logging in and searching for relevant information [71].

We identified only 4 records that objectively measured the cost-effectiveness of patients’ data management. A retrospective cross-sectional study investigating the impact of patients updating their medication list via a secure message feature showed that its use did not significantly decrease the cost burden of outpatient clinics [58]. However, another retrospective study found that asking patients to review and update their medical history via a computer terminal kiosk in the waiting room of a chemotherapy clinic reduced the medication reconciliation time by nearly 50% [51]. A retrospective longitudinal cohort study also found that active portal users were less likely to contact or visit their health care providers [61], whereas another retrospective analysis of portal use showed that nonusers visited the emergency room more often than active users, even though active users had more outpatient and inpatient visits [62].

## Discussion

### Principal Findings

This synthesis of literature explored the barriers and facilitators that patients face when they decide to generate and manage their core medical data in (tools linked to) their PEHRs. First, we found that a minority of registered users entered, updated, or modified their personal and medical data. More specifically, less than half of the registered users entered their data and less than a quarter of users updated or modified their already recorded data; continued use further dropped to <10% of the user population as time increased. Patients preferred to take on a passive rather than an active role regarding the self-management of their health information, and they seemed to prefer tracking vital signs above more complex medical information, such as medications and their family health history. We identified both patients’ and health care professionals’ (positive) perceptions about the validity, applicability, and confidentiality of patient-generated data as well as patients’ digital and health literacy as important facilitators of patients’ active management of their personal and medical data. However, we also found that patients’ and health care providers’ concerns about the validity and applicability of patient-generated data seem to be unfounded. Patients accurately reported on their diagnoses, medications, immunizations, medical history, and family health history, making their medical records more complete. Moreover, patients who managed their medical data felt more knowledgeable, more in control of their own health care, and more adherent to their treatment than less active patients. Both patients and clinicians felt that active patients were also more prepared for their clinical visits because they knew which questions they wanted answered by their health care provider. In the following sections, we propose recommendations that health care practices can adopt for stimulating patient participation in the generation and management of their electronic core medical data.

### The Health Care Provider as Ambassador and Gatekeeper

Patients felt that they were bypassing clinical staff when they self-managed their medical data. Patients were concerned that they would provide their physicians with inaccurate information, especially when the nature of the medical information is complex and sensitive. Clear guidelines and information regarding the added value of patient-entered data for both patients and clinicians may reduce these concerns. Clinical staff are important ambassadors for informing their patients about the added value of patient-generated and management data and in reminding and encouraging their patients to prepare themselves for each visit by reviewing the medical data in their PEHRs. Moreover, we also found that self-management of medical data may be higher for those patients who feel that they are able to directly contact their provider for support. Design features within the PEHR systems that amplify the visibility of the health care providers’ availability for support and guidance as well as visual feedback elements in the PEHR system that indicate to the patients that their entered or modified data will be checked by a professional may reassure patients that they are not altering their medical record without their provider’s knowledge or approval.

### Ethical and Comprehensive by Design

We also found that patients were generally concerned that their medical data were unprotected against unauthorized access and could, therefore, be used for non–health care–related purposes. Stressing data confidentiality and allowing patients to give their informed consent on an opt-in and opt-out basis may diminish their potential unease about confidentiality. Furthermore, we have also seen that customization features may enhance the self-management of core medical data because they make the system more understandable and easier to use. Helping patients to remember medical information by using prepopulated forms or guided data entry might further aid and encourage them to record information that might be inaccurate. This may also address health care providers’ concerns that patients are not able to accurately report on their medical information.

### Future Directions

On the basis of our findings and recommendations, we have outlined several priority questions for future studies (Textbox 1) that we address briefly in this section. The first 2 questions are related to the finding that health care providers play an important role in their patients’ uptake and continued use of (tools linked to) their PEHRs to manage their core medical data. It is still not known what providers need for addressing their concerns about the validity and applicability of patient-generated data. Thus, we invite future studies to explore the needs of professionals in terms of (portal) assistance or (system) requirements so that they are willing to encourage the practice of patients’ self-management medical data and their patients feel stimulated and supported to manage their core medical data during their entire care journey as a result.

**Priority questions for future research based on our 3 recommendations.**

1. The health care provider as ambassador and gatekeeper

- What are the unmet needs of health care professionals with respect to encouraging and supporting their patients to share and manage their personal and medical data during their care journey?
- What are the unmet needs of patients in terms of feeling encouraged and supported by their health care providers to share and manage their personal and medical data during their care journey?

2. Ethical and comprehensive by design

- What do patients need in terms of assistance, support, and system requirements, to generate and manage their personal data during their care journey?
- To what extent does the type of personal and medical data affect patients’ data management?

3. Stimulating the patient-provider partnership

- When do patients consider themselves to be “active” managers of their personal and medical data, and to what extent does this correspond to health care professionals’ perspectives?
- To what extent do patients’ perspectives on their personal data management activity and role preference affect their data management?

For fear of reporting inadequate information, patients prefer to report their core medical data in a structured, guided manner. Our review showed that this was the case for data that were perceived to be error-prone and sensitive, such as information about the types, names, and dosages of patients’ medications or information about patients’ family health history that would be used for genetic counseling. This finding corresponds to the findings of Esmaeilzadeh et al [73], who showed that individuals were more willing to share sensitive and private information about their mental or physical illnesses when they could enter this information by following a structured, organized, and predefined data entry model, as opposed to using an unstructured, text-heavy interface [73]. Taken together, this seems to indicate that guided data entry interfaces may stimulate patients to share personal health information they would not otherwise share because they do not feel confident or knowledgeable enough to share it or because confidentiality or privacy concerns prevent them from doing so. However, we also found that in case of sensitive information, patients may feel that closed answer options do not offer sufficient granularity and feel the need to add additional contextual information to their answers. Hence, we invite future studies to explore the extent to which patients’ preference for structured data entry models is dependent on the type of data that they wish to record.

We have also shown that patients prefer to update and monitor data about their vital signs (eg, blood glucose levels and BMI) over updating information about their medications, allergies, intoxications, and social and family history. To the best of our knowledge, no studies to date have examined the reasons for these differences. On the basis of the findings of our review, we hypothesize that patients prefer to manage data about their vital signs to managing information about other core medical data because they are trackable over time and thereby give patients a more direct, visible insight into their health status compared with other core medical data. We encourage future studies to explore this explanation.

We have shown that the number of studies that focus on actual portal use—by exploring how patients use their portal, whether and when patients consider themselves to be active users, which data patients share, and how frequently they do this—remains scarce. Interestingly, it is not common practice for patient data management papers to describe in full detail whether, how, and how frequently and what type of medical information is entered, updated, or modified by patients. We believe that this is mainly caused by an undifferentiated definition of the term “active user.” In the retrieved literature, users were predominantly considered to be active based solely on whether they activated their account [74], the number of times they logged in or accessed a certain page or implemented tool [75], or their self-reported (undefined and abstract) use of the portal [76]. Patients were described to be active when they performed an activity once [40,42,53,56-58,65,67,70], more than once [49], >3 times [38], >20 times [61], or more than once every 4 months [62]. It would be a promising endeavor for future research to define “active data management” from both the patients’ and their care professionals’ perspectives.

Our findings are in line with research that has investigated the extent to which patients participate in making decisions together with their physicians regarding treatment plans. Shared decision-making entails the collaborative exchange and discussion of health care information among patients and their health care providers, including information about patient preferences and the pros and cons of all possible treatment options [77,78]. Collaboration is the key here [79], meaning that both patients and health care providers are jointly responsible for reducing asymmetries in information exchange so that treatment decisions that patients can adhere to because they optimally align with their wishes and abilities are reached [80]. One line of research claims that not all patients have the desire to participate in decision-making processes [80-82] and that this is especially the case for older and less healthy patients who, ironically, might benefit the most from being involved [83]. Another line of research claims that most patients do in fact want to be informed and involved, but that they cannot fulfill this desire because it is not acknowledged or afforded to them by their health care provider [80,84]. Patients’ preferred and assumed roles often do not match [85], leading to decisional role regret [86]. In many cases, physicians do not know their patients well enough. Patients believe that the medical expertise and knowledge of their health care provider are more important than their own knowledge and preferences. Thus, our advice is to inform patients about the complementary value that they bring to the shared decision-making process and to improve patients’ confidence in their capability to acquire and understand the information that is necessary to make informed decisions based on the available options [84,87]. Our literature review showed that these recommendations also apply when clinical staff want to involve patients in the management of their medical data. We invite future studies to explore the extent to which discrepancies in patients’ preferred versus assumed roles in the management of their medical data affect their engagement and satisfaction with their clinical care.

### Limitations

This scoping review has some limitations. We retrieved a limited set of highly heterogeneous papers because they provided detailed information about patients’ actual data management activities. Despite the considerable heterogeneity in the study objectives, designs, and outcome measures used in these papers, we were able to identify key themes regarding the facilitators and barriers that patients face when they decide to generate and manage their medical data. In addition, this review concentrated on measurable uses of PEHRs (ie, entering, updating, and modifying data) to identify what stimulates or prevents patients’ use. Although patients who evaluate their core medical data and subsequently decide not to add or modify information are actively engaging with their PEHR, we chose not to include this group because we would then need to rely on log-in frequencies to determine the patients’ (level of) engagement with their health data. Not only may log-in frequencies be biased by false log-in data resulting from log-in problems, but they also do not inform us whether a log-in moment resulted in meaningful use of the portal. A promising endeavor for future studies would be to identify whether and how frequently patients review and approve of the core medical data recorded in their PEHR and which factors contribute to this type of use.

### Conclusions

Most patients do not actively review and enter, update, or modify their medical data in a PEHR. Patients refrain from generating and managing their medical data, especially when medical information is complex and sensitive. The reasons for patients’ passive behavior are their concerns about the validity, applicability, and confidentiality of patient-generated data, although we found that patient-generated data are often accurate and helpful in stimulating patient engagement and satisfaction. We have offered recommendations for implementing design features within the (tools linked to) PEHRs and the creation of a dedicated policy to inform both clinical staff and patients about the added value of patient-generated data, with clinicians being involved as important ambassadors in informing, reminding, and encouraging patients to manage the data in their PEHR.

## Appendix

Multimedia Appendix 1 Filled-in PRISMA-ScR (Preferred Reporting Items for Systematic Reviews and Meta-Analyses Extension for Scoping Reviews) checklist [31].

Multimedia Appendix 2 Search strategy for the MEDLINE, PsycINFO, CINAHL, Cochrane Library, Embase, Web of Science, and Google Scholar databases.

Multimedia Appendix 3 Data extraction form.

## Abbreviations

PEHR: personal electronic health record
PRISMA-ScR: Preferred Reporting Items for Systematic Reviews and Meta-Analyses Extension for Scoping Reviews
