# Supplementary material for: Patients Managing Their Medical Data in Personal Electronic Health Records: Scoping Review
Source: J Med Internet Res. 2022 Dec 27;24(12):e37783. doi: 10.2196/37783 (PMC9832357; doi:10.2196/37783)
Supplement: Multimedia Appendix 2 [file jmir_v24i12e37783_app2.pdf]

Multimedia Appendix 2

Table 1. Search strategy MEDLINE (ran through Pubmed).

1	"Medical Records Systems, Computerized" [Mesh: NoExp]
2	Electronic Health Records [MeSH Terms]
3	Health Records, Personal [MeSH Terms]
4	("Medical Informatics/education"[Mesh] OR "Medical Informatics/instrumentation"[Mesh] OR "Medical Informatics/methods"[Mesh] OR "Medical Informatics/nursing"[Mesh] OR "Medical Informatics/organization and administration"[Mesh] OR "Medical Informatics/psychology"[Mesh] OR "Medical Informatics/therapy"[Mesh])
5	User-Computer Interface [MeSH Terms]
7	Patient Access to Records [Mesh Terms]
8	"electronic health record*" [tiab] OR "electronic medical record*" [tiab] OR emr [tiab] OR ehr [tiab] OR "electronic patient record*" [tiab] OR "online health record*" [tiab] OR "online medical record*" [tiab] OR "online patient record*" [tiab] OR "personal health record*" [tiab] OR "personal medical record*" [tiab]
9	"patient reported health" [tiab] OR "patient reported medical" [tiab] OR "patient reported outcome*" [tiab] OR "patient generated health" [tiab] OR "patient generated medical" [tiab]
10	"patient portal*" [tiab]
11	mychart [tiab] OR "electronic health service*" [tiab] OR "web portal*" [tiab]
12	OR / #1 - #11
13	Patient Participation [MeSH Terms]
14	Empowerment [MeSH Terms]
15	Patient Reported Outcome Measures [MeSH: NoExp]
16	Decision Making [Mesh: NoExp]
17	Clinical Decision-Making/methods [MeSH Terms]
18	Decision Making, Shared [MeSH Terms]
19	Choice Behavior [Mesh: NoExp]
20	Patient-Centered Care [Mesh: NoExp]
21	("Self Care/education"[Mesh] OR "Self Care/instrumentation"[Mesh] OR "Self Care/methods"[Mesh] OR "Self Care/nursing"[Mesh] OR "Self Care/organization and administration"[Mesh] OR "Self Care/psychology"[Mesh] OR "Self Care/therapy"[Mesh])
22	("Self-Management/education"[Mesh] OR "Self-Management/methods"[Mesh] OR "Self-Management/organization and administration"[Mesh] OR "Self-Management/psychology"[Mesh])
23	Consumer Health Information [MeSH Terms]
24	Patient Education as Topic [Mesh: NoExp]
25	Health Knowledge, Attitudes and Practice [MeSH Terms]
26	("Disease Management/instrumentation"[Mesh] OR "Disease Management/methods"[Mesh] OR "Disease Management/nursing"[Mesh] OR "Disease Management/organization and administration"[Mesh] OR "Disease Management/therapy"[Mesh])
27	("Quality of Health Care/education"[Mesh] OR "Quality of Health Care/instrumentation"[Mesh] OR "Quality of Health Care/methods"[Mesh] OR "Quality of Health Care/nursing"[Mesh] OR "Quality of Health Care/organization and administration"[Mesh] OR "Quality of Health Care/prevention and control"[Mesh] OR "Quality of Health Care/psychology"[Mesh] OR "Quality of Health Care/therapy"[Mesh])
28	("Quality of Life/organization and administration"[Mesh] OR "Quality of Life/psychology"[Mesh])
29	Patient Satisfaction [MeSH Terms]
30	Attitude of Health Personnel [Mesh: NoExp]
31	Therapeutic Alliance [MeSH Terms]
32	Patient Safety [MeSH Terms]
33	Health information management [MeSH Terms]
34	"self monitoring" [tiab] OR "self management" [tiab] OR "self care" [tiab] OR "self regulation" [tiab]
35	"patient centered care" [tiab] OR "client centered care" [tiab] OR "person centered care" [tiab] OR "patient focused care" [tiab] OR "client focused care" [tiab] OR "person focused care" [tiab] OR "patient centered healthcare" [tiab] OR "client centered healthcare" [tiab] OR "person centered healthcare" [tiab]

	OR “patient focused healthcare” [tiab] OR “client focused healthcare” [tiab] OR “person focused healthcare” [tiab]
36	“patient engagement” [tiab] OR “patient participation” [tiab] OR “patient involvement” [tiab] OR “consumer engagement” [tiab] OR “patient activation” [tiab] OR “consumer activation” [tiab] OR “consumer participation” [tiab] OR “consumer involvement” [tiab] OR “client engagement” [tiab] OR “client participation” [tiab] OR “client involvement” [tiab] OR “client activation” [tiab] OR “patient education” [tiab] OR “patient decision making” [tiab] OR “shared decision making” [tiab]
37	OR / #13 - #36
38	Medication Reconciliation [MeSH Terms]
39	Medical History Taking [Mesh: NoExp]
40	Medical Records, Problem-Oriented [MeSH Terms]
41	Immunization [Mesh: NoExp]
42	Vaccination [Mesh: NoExp]
43	("Chronic Disease/methods"[Mesh] OR "Chronic Disease/nursing"[Mesh] OR "Chronic Disease/organization and administration"[Mesh] OR "Chronic Disease/prevention and control"[Mesh] OR "Chronic Disease/psychology"[Mesh] OR "Chronic Disease/therapy"[Mesh])
44	“portal use” [tiab] OR “portal usage” [tiab]
45	“personal health information” [tiab] OR “personal medical information” [tiab] OR “patient health information” [tiab] OR “patient medical information” [tiab] OR “consumer health information” [tiab] OR “consumer medical information” [tiab]
46	“problem list” [tiab] OR “problem diagnosis” [tiab] OR “medication regimen” [tiab] OR “medical regimen” [tiab] OR “medical history” [tiab] OR “medication history” [tiab] OR “patient history” [tiab] OR “family history” [tiab] OR “medication reconciliation” [tiab] OR “medication verification” [tiab] OR vaccin* [tiab] OR allerg* [tiab] OR immunization* [tiab] OR immunisation* [tiab] OR intoxication* [tiab] OR poisoning [tiab]
47	OR / #38 - #46
48	12 AND 37 AND 47
49	Limit 48 to (English or Dutch language and yr=2000-2020)

Note. We ran this search strategy through MEDLINE (PubMed) again in March 2022, retrieving studies published from April 2020 till March 2022.

Table 2. Search strategy PsycINFO.

1	MA Electronic Health Records
2	MA Client Records
3	MA Human Computer Interaction OR MA Human Computer Interaction Measures
4	MA Electronic Health Services
5	MA Health Information Technology
6	TI ("electronic health record*" OR "electronic medical record*" OR emr OR ehr OR "electronic patient record*" OR "online health record*" OR "online medical record*" OR "online patient record*" OR "personal health record*" OR "personal medical record*") OR AB ("electronic health record*" OR "electronic medical record*" OR emr OR ehr OR "electronic patient record*" OR "online health record*" OR "online medical record*" OR "online patient record*" OR "personal health record*" OR "personal medical record*")
7	TI ("patient reported health" OR "patient reported medical" OR "patient reported outcome*" OR "patient generated health" OR "patient generated medical") OR AB ("patient reported health" OR "patient reported medical" OR "patient reported outcome*" OR "patient generated health" OR "patient generated medical")
8	TI (patient portal*) OR AB (patient portal*)
9	TI (mychart OR "electronic health service*" OR "web portal*") OR AB (mychart OR "electronic health service*" OR "web portal*")
10	OR / #1 - #9
11	MA Client Participation
12	MA Empowerment
13	MA Patient Reported Outcome Measures
14	MA Decision Making
15	MA Choice Behavior
16	MA Self-Management
17	MA Self-Monitoring
18	MA Client Education
19	MA Health Knowledge
20	MA Disease Management
21	MA Quality of Care
22	MA Client Attitudes
23	MA Client Satisfaction
24	MA Health Attitudes
25	MA Health Personnel Attitudes
26	MA Therapeutic Alliance
27	MA Quality of Life
28	MA Patient Safety
29	MA Electronic Health Services
30	MA Digital Interventions
31	TI ("self monitoring" OR "self management" OR "self care" OR "self regulation") OR AB ("self monitoring" OR "self management" OR "self care" OR "self regulation")
32	TI ("patient centered care" OR "client centered care" OR "person centered care" OR "patient focused care" OR "client focused care" OR "person focused care" OR "patient centered healthcare" OR "client centered healthcare" OR "person centered healthcare" OR "patient focused healthcare" OR "client focused healthcare" OR "person focused healthcare") OR AB ("patient centered care" OR "client centered care" OR "person centered care" OR "patient focused care" OR "client focused care" OR "person focused care" OR "patient centered healthcare" OR "client centered healthcare" OR "person centered healthcare" OR "patient focused healthcare" OR "client focused healthcare" OR "person focused healthcare")
33	TI ("patient engagement" OR "patient participation" OR "patient involvement" OR "consumer engagement" OR "patient activation" OR "consumer activation" OR "consumer participation" OR "consumer involvement" OR "client engagement" OR "client participation" OR "client involvement" OR "client activation" OR "patient education" OR "patient decision making" OR "shared decision making") OR AB ("patient engagement" OR "patient participation" OR "patient involvement" OR "consumer engagement" OR "patient activation" OR "consumer activation" OR "consumer

	participation" OR "consumer involvement" OR "client engagement" OR "client participation" OR "client involvement" OR "client activation" OR "patient education" OR "patient decision making" OR "shared decision making")
34	OR / #11 - #33
35	MA Patient history
36	MA Family history
37	MA Immunization
38	MA Toxic Disorders
39	MA Chronic Illness
40	TI ("portal use" OR "portal usage") OR AB ("portal use" OR "portal usage")
41	TI ("personal health information" OR "personal medical information" OR "patient health information" OR "patient medical information" OR "consumer health information" OR "consumer medical information") OR AB ("personal health information" OR "personal medical information" OR "patient health information" OR "patient medical information" OR "consumer health information" OR "consumer medical information")
42	TI ("problem list" OR "problem diagnosis" OR "medication regimen" OR "medical regimen" OR "medical history" OR "medication history" OR "patient history" OR "family history" OR "medication reconciliation" OR "medication verification" OR vaccin* OR allerg* OR immunization* OR immunisation* OR intoxication* OR poisoning) OR AB ("problem list" OR "problem diagnosis" OR "medication regimen" OR "medical regimen" OR "medical history" OR "medication history" OR "patient history" OR "family history" OR "medication reconciliation" OR "medication verification" OR vaccin* OR allerg* OR immunization* OR immunisation* OR intoxication* OR poisoning)
43	OR / #35 - #42
44	10 AND 34 AND 43
45	Limit 44 to (English or Dutch language and yr=2000-2020)

Table 3. Search strategy CINAHL (Plus with full text).

1	MH Patient Record System
2	MH Electronic Health Records+
3	MH Medical Records, Personal
4	(MH "Medical Informatics/AM/ED/ES/EV/MT/UT/ST")
5	(MH "Health Informatics/AM/ED/ES/EV/MT/UT/ST")
6	MH User-Computer Interface
7	MH Patient Access to Records
8	TI (“electronic health record*” OR “electronic medical record*” OR emr OR ehr OR “electronic patient record*” OR “online health record*” OR “online medical record*” OR “online patient record*” OR “personal health record*” OR “personal medical record*”) OR AB (“electronic health record*” OR “electronic medical record*” OR emr OR ehr OR “electronic patient record*” OR “online health record*” OR “online medical record*” OR “online patient record*” OR “personal health record*” OR “personal medical record*”)
9	TI (“patient reported health” OR “patient reported medical” OR “patient reported outcome*” OR “patient generated health” OR “patient generated medical”) OR AB (“patient reported health” OR “patient reported medical” OR “patient reported outcome*” OR “patient generated health” OR “patient generated medical”)
10	TI (“patient portal*”) OR AB (“patient portal*”)
11	TI (mychart OR “electronic health service*” OR “web portal*”) OR AB (mychart OR “electronic health service*” OR “web portal*”)
12	OR / #1 - #11
13	MH Consumer Participation
14	MH Empowerment
15	MH Patient Reported Outcomes
16	MH Decision Making
17	MH "Decision Making, Clinical/MT"
18	MH Decision Making, Shared
19	MH Decision Making, Patient
20	MH Patient Centered Care
21	(MH "Self Care/ED/ES/EV/MT/NU/PF/ST/UT")
22	(MH "Self-Management/ED/ES/EV/MT/NU/PF/ST/UT")
23	MH Consumer Health Information+
24	MH Patient Education
25	MH Health Knowledge
26	(MH "Disease Management/AM/ED/EV/MT/UT/ST")
27	(MH "Quality of Health Care/AM/ED/EV/ST/UT")
28	(MH "Quality of Life/ED/EV/PC/PF/ST/UT")
29	MH Patient Satisfaction+
30	MH Patient Attitudes
31	MH Caregiver Attitudes
32	MH Attitude of Health Personnel+
33	MH Therapeutic Alliance
34	MH Patient Safety
35	MH Health Information Management
36	TI (“self monitoring” OR “self management” OR “self care” OR “self regulation”) OR AB (“self monitoring” OR “self management” OR “self care” OR “self regulation”)
37	TI (“patient centered care” OR “client centered care” OR “person centered care” OR “patient focused care” OR “client focused care” OR “person focused care” OR “patient centered healthcare” OR “client centered healthcare” OR “person centered healthcare” OR “patient focused healthcare” OR “client focused healthcare” OR “person focused healthcare”) OR AB (“patient centered care” OR “client centered care” OR “person centered care” OR “patient focused care” OR “client focused care” OR “person focused care” OR “patient centered healthcare” OR “client centered healthcare” OR “person centered healthcare” OR “patient focused healthcare” OR “client focused healthcare” OR “person focused healthcare”)

38	TI (“patient engagement” OR “patient participation” OR “patient involvement” OR “consumer engagement” OR “patient activation” OR “consumer activation” OR “consumer participation” OR “consumer involvement” OR “client engagement” OR “client participation” OR “client involvement” OR “client activation” OR “patient education” OR “patient decision making” OR “shared decision making”) OR AB (“patient engagement” OR “patient participation” OR “patient involvement” OR “consumer engagement” OR “patient activation” OR “consumer activation” OR “consumer participation” OR “consumer involvement” OR “client engagement” OR “client participation” OR “client involvement” OR “client activation” OR “patient education” OR “patient decision making” OR “shared decision making”)
39	OR / #13 - #38
40	MH Medication Reconciliation
41	MH Patient History Taking+
42	MH Problem Oriented Records
43	MH Immunization
44	(MH "Chronic Disease/ED/EV/MT/NU/PC/PF/ST/TH/UT")
45	TI (“portal use” OR “portal usage”) OR AB (“portal use” OR “portal usage”)
46	TI (“personal health information” OR “personal medical information” OR “patient health information” OR “patient medical information” OR “consumer health information” OR “consumer medical information”) OR AB (“personal health information” OR “personal medical information” OR “patient health information” OR “patient medical information” OR “consumer health information” OR “consumer medical information”)
47	TI (“problem list” OR “problem diagnosis” OR “medication regimen” OR “medical regimen” OR “medical history” OR “medication history” OR “patient history” OR “family history” OR “medication reconciliation” OR “medication verification” OR vaccin* OR allerg* OR immunization* OR immunisation* OR intoxication* OR poisoning) OR AB (“problem list” OR “problem diagnosis” OR “medication regimen” OR “medical regimen” OR “medical history” OR “medication history” OR “patient history” OR “family history” OR “medication reconciliation” OR “medication verification” OR vaccin* OR allerg* OR immunization* OR immunisation* OR intoxication* OR poisoning)
48	OR / #40 - #47
49	#12 AND #39 AND #48
50	Limit 49 to (English or Dutch language and yr=2000-2020)

Table 3. Search strategy Cochrane Library.

1	MeSH descriptor: [Medical Records Systems, Computerized] this term only
2	MeSH descriptor: [Electronic Health Records] this term only
3	MeSH descriptor: [Health Records, Personal] explode all trees
4	MeSH descriptor: [Medical Informatics] explode all trees and with qualifier(s): [education - ED, instrumentation - IS, methods - MT, organization & administration - OG, standards - ST]
5	MeSH descriptor: [Health Information Exchange] this term only
6	MeSH descriptor: [User-Computer Interface] explode all trees
7	MeSH descriptor: [Patient Access to Records] this term only
8	(“electronic health record*”):ti,ab,kw OR (“electronic medical record*”):ti,ab,kw OR (emr):ti,ab,kw OR (ehr):ti,ab,kw OR (“electronic patient record*”):ti,ab,kw OR (“online health record*”):ti,ab,kw OR (“online medical record*”):ti,ab,kw OR (“online patient record*”):ti,ab,kw OR (“personal health record*”):ti,ab,kw OR (“personal medical record*”):ti,ab,kw (Word variations have been searched)
9	(“patient reported health”):ti,ab,kw OR (“patient reported medical”):ti,ab,kw OR (“patient reported outcome*”):ti,ab,kw OR (“patient generated health”):ti,ab,kw OR (“patient generated medical”):ti,ab,kw (Word variations have been searched)
10	("patient portal*"):ti,ab,kw (Word variations have been searched)
11	("mychart"):ti,ab,kw OR ("electronic health service"):ti,ab,kw OR ("web portal*"):ti,ab,kw (Word variations have been searched)
12	OR / #1 - #11
13	MeSH descriptor: [Patient Participation] this term only]
14	MeSH descriptor: [Empowerment] this term only
15	MeSH descriptor: [Patient Reported Outcome Measures] this term only
16	MeSH descriptor: [Decision Making] this term only
17	MeSH descriptor: [Clinical Decision-Making] explode all trees and with qualifier(s): [methods - MT]
18	MeSH descriptor: [Choice Behavior] this term only
9	MeSH descriptor: [Decision Making, Shared] this term only
20	MeSH descriptor: [Patient-Centered Care] this term only
21	MeSH descriptor: [Self Care] this term only and with qualifier(s): [instrumentation - IS, methods - MT, psychology - PX, standards - ST]
22	MeSH descriptor: [Self-Management] this term only and with qualifier(s): [education - ED, methods - MT, psychology - PX]
23	MeSH descriptor: [Consumer Health Information] explode all trees
24	MeSH descriptor: [Patient Education as Topic] this term only
25	MeSH descriptor: [Disease Management] this term only
26	MeSH descriptor: [Quality of Health Care] this term only and with qualifier(s): [organization & administration - OG, standards - ST]
27	MeSH descriptor: [Patient Satisfaction] explode all trees
28	MeSH descriptor: [Health Knowledge, Attitudes, Practice] this term only
29	MeSH descriptor: [Therapeutic Alliance] this term only
30	MeSH descriptor: [Attitude of Health Personnel] this term only
31	MeSH descriptor: [Quality of Life] this term only and with qualifier(s): [psychology - PX]
32	MeSH descriptor: [Patient Safety] this term only
33	MeSH descriptor: [Health Information Management] explode all trees
34	(“self monitoring”:ti,ab,kw OR (“self management”):ti,ab,kw OR (“self care”):ti,ab,kw OR (“self regulation”):ti,ab,kw (Word variations have been searched)
35	(“patient centered care”):ti,ab,kw OR (“client centered care”):ti,ab,kw OR (“person centered care”):ti,ab,kw OR (“patient focused care”):ti,ab,kw OR (“client focused care”):ti,ab,kw OR (“person focused care”):ti,ab,kw OR (“patient centered healthcare”):ti,ab,kw OR (“client centered healthcare”):ti,ab,kw OR (“person centered healthcare”):ti,ab,kw OR (“patient focused healthcare”):ti,ab,kw OR (“client focused healthcare”):ti,ab,kw OR (“person focused healthcare”):ti,ab,kw (Word variations have been searched)
36	(“patient engagement”):ti,ab,kw OR (“patient participation”):ti,ab,kw OR (“patient involvement”):ti,ab,kw OR (“consumer engagement”):ti,ab,kw OR (“patient activation”):ti,ab,kw OR (“consumer activation”):ti,ab,kw OR (“consumer participation”):ti,ab,kw OR (“consumer involvement”):ti,ab,kw OR (“client engagement”):ti,ab,kw OR (“client participation”):ti,ab,kw OR

	(“client involvement”):ti,ab,kw OR (“client activation”):ti,ab,kw OR (“patient education”):ti,ab,kw OR (“patient decision making”):ti,ab,kw OR (“shared decision making”):ti,ab,kw (Word variations have been searched)
37	OR / #13 - #36
38	MeSH descriptor: [Medication Reconciliation] this term only
39	MeSH descriptor: [Medical History Taking] this term only
40	MeSH descriptor: [Medical Records, Problem-Oriented] this term only
41	MeSH descriptor: [Immunization] this term only
42	MeSH descriptor: [Vaccination] this term only
43	MeSH descriptor: [Chronic Disease] this term only and with qualifier(s): [nursing - NU, prevention & control - PC, psychology - PX, rehabilitation - RH, therapy - TH]
44	("portal use"):ti,ab,kw OR ("portal usage"):ti,ab,kw (Word variations have been searched)
45	("personal health information"):ti,ab,kw OR (“personal medical information”):ti,ab,kw OR (“patient health information”):ti,ab,kw OR (“patient medical information”):ti,ab,kw OR (“consumer health information”):ti,ab,kw OR (“consumer medical information”):ti,ab,kw (Word variations have been searched)
46	(“problem list”):ti,ab,kw OR (“problem diagnosis”):ti,ab,kw OR (“medication regimen”):ti,ab,kw OR (“medical regimen”):ti,ab,kw OR (“medical history”):ti,ab,kw OR (“medication history”):ti,ab,kw OR (“patient history”):ti,ab,kw OR (“family history”):ti,ab,kw OR (“medication reconciliation”):ti,ab,kw OR (“medication verification”):ti,ab,kw OR (vaccin*):ti,ab,kw OR (allerg*):ti,ab,kw OR (immunization*):ti,ab,kw OR (immunisation*):ti,ab,kw OR (intoxication*):ti,ab,kw OR (poisoning):ti,ab,kw (Word variations have been searched)
47	OR / #38 - #46
48	#12 AND #37 AND #47
49	Limit 48 to (English or Dutch language and yr=2000-2020)

Table 5. Search Strategy EMBASE.

1	medical record/
2	exp electronic health record/
3	exp electronic medical record/
4	electronic patient record/
5	exp medical informatics/
6	exp medical information system/
7	exp computer interface/
8	exp patient right
9	exp human computer interaction/
10	(electronic health record*).ab,ti OR (electronic medical record*).ab,ti OR emr.ab,ti OR ehr.ab,ti OR (electronic patient record*).ab,ti OR (online health record*).ab,ti OR (online medical record*).ab,ti OR (online patient record*).ab,ti OR (personal health record*).ab,ti OR (personal medical record*).ab,ti
11	(patient reported health).ab,ti OR (patient reported medical).ab,ti OR (patient reported outcome*****).ab,ti OR (patient generated health).ab,ti OR (patient generated medical) .ab,ti
12	(patient portal*).ab,ti
13	mychart.ab,ti OR (electronic health service*).ab,ti OR (web portal*).ab,ti)
14	OR / #1 - #13
15	patient-reported outcome/
16	patient participation/ OR exp empowerment/ OR exp health literacy/
17	self care/
18	exp patient education/ OR exp consumer health information OR disease management/
19	patient care/
20	decision making/ OR exp shared decision making/ OR exp patient decision making/ OR exp family decision making/ OR clinical decision making/
21	health care quality/
22	exp patient satisfaction/ OR exp attitude to health OR exp therapeutic alliance/ OR health personnel attitude/ OR exp patient preference/
23	exp self monitoring/
24	exp patient safety/
25	(self monitoring).ab,ti OR (self management).ab,ti OR (self care).ab,ti OR (self regulation).ab,ti
26	(patient centered care).ab,ti OR (client centered care).ab,ti OR (person centered care).ab,ti OR (patient focused care).ab,ti OR (client focused care).ab,ti OR (person focused care).ab,ti OR (patient centered healthcare).ab,ti OR (client centered healthcare).ab,ti OR (person centered healthcare).ab,ti OR (patient focused healthcare).ab,ti OR (client focused healthcare).ab,ti OR (person focused healthcare).ab,ti

27	(patient engagement).ab,ti OR (patient participation).ab,ti OR (patient involvement).ab,ti OR (patient activation).ab,ti OR (consumer engagement).ab,ti OR (consumer activation).ab,ti OR (consumer participation).ab,ti OR (consumer involvement).ab,ti OR (client engagement).ab,ti OR (client participation).ab,ti OR (client involvement).ab,ti OR (client activation).ab,ti OR (patient education).ab,ti OR (patient decision making).ab,ti OR (shared decision making).ab,ti
28	OR / #15 - #27
29	exp medication therapy management/
30	medical history/
31	immunization/ OR vaccination/
32	exp consumer health information/
33	exp family history
34	chronic disease/dm, pc, th
35	(portal us*).ab,ti
36	(personal health information).ab,ti OR (personal medical information).ab,ti OR (patient health information).ab,ti OR (patient medical information).ab,ti OR (consumer health information).ab,ti OR (consumer medical information).ab,ti
37	(problem list).ab,ti OR (problem diagnosis).ab,ti OR (medication regimen).ab,ti OR (medical regimen).ab,ti OR (medical history).ab,ti OR (medication history).ab,ti OR (patient history).ab,ti OR (family history).ab,ti OR (medication reconciliation).ab,ti OR (medication verification).ab,ti OR vaccin*.ab,ti OR allerg*.ab,ti OR immunization.ab,ti OR immunisation.ab,ti OR intoxication.ab,ti OR poisoning.ab,ti
38	29 OR 30 OR 31 OR 32 OR 33 OR 34 OR 35 OR 36 OR 37
39	#14 AND #28 AND #38
40	Limit 39 to (exclude medline journals and embase and (dutch or english) and yr=”2000-Current”)

Table 6. Search strategy Web of Science.

1	TS = ("electronic health record*" OR "electronic medical record*" OR emr OR ehr OR "electronic patient record*" OR "online health record*" OR "online medical record*" OR "online patient record*" OR "personal health record*" OR "personal medical record*" OR "patient reported health" OR "patient reported medical" OR "patient reported outcome*" OR "patient generated health" OR "patient generated medical" OR "patient portal*" OR mychart OR "electronic health service*" OR "web portal*")
2	TS=("self monitoring" OR "self management" OR "self care" OR "self regulation" OR "patient centered care" OR "client centered care" OR "person centered care" OR "patient focused care" OR "client focused care" OR "person focused care" OR "patient centered healthcare" OR "client centered healthcare" OR "person centered healthcare" OR "patient focused healthcare" OR "client focused healthcare" OR "person focused healthcare" OR "patient engagement" OR "patient participation" OR "patient involvement" OR "consumer engagement" OR "patient activation" OR "consumer activation" OR "consumer participation" OR "consumer involvement" OR "client engagement" OR "client participation" OR "client involvement" OR "client activation" OR "patient education" OR "patient decision making" OR "shared decision making")
3	TS=("portal use" OR "portal usage" OR "personal health information" OR "personal medical information" OR "patient health information" OR "patient medical information" OR "consumer health information" OR "consumer medical information" OR "problem list" OR "problem diagnosis" OR "medication regimen" OR "medical regimen" OR "medical history" OR "medication history" OR "patient history" OR "family history" OR "medication reconciliation" OR "medication verification" OR vaccin* OR allerg* OR immunization* OR immunisation* OR intoxication* OR poisoning)
4	#1 AND #2 AND #3
5	Limit #4 to (English or Dutch language and yr=2000-2020)

Table 7. Search strategy Google Scholar #1.

1	With all of the words:	electronic health record
2	With the exact phrase:	
3	With at least one of the words:	doctor physician clinician professional giver provider patient portal engagement participation involvement "portal use" "portal usage"
4	Results in:	allintitle: electronic health record giver OR provider OR doctor OR physician OR clinician OR professional OR patient OR portal OR engagement OR participation OR involvement OR "portal use" OR "portal usage"
5	Return articles dated between:	2000-2020 (exclude citations and patents)

Table 8. Search strategy Google Scholar #2.

1	With all of the words:	electronic medical record
2	With the exact phrase:	
3	With at least one of the words:	doctor physician clinician professional giver provider patient portal engagement participation involvement "portal use" "portal usage"
4	Results in:	allintitle: electronic medical record giver OR provider OR doctor OR physician OR clinician OR professional OR patient OR portal OR engagement OR participation OR involvement OR "portal use" OR "portal usage"
5	Return articles dated between:	2000-2020 (exclude citations and patents)

Table 9. Search strategy Google Scholar #3.

1	With all of the words:	
2	With the exact phrase:	patient portal
3	With at least one of the words:	doctor physician clinician professional giver provider patient portal engagement participation involvement "portal use" "portal usage"
4	Results in:	allintitle: giver OR provider OR doctor OR physician OR clinician OR professional OR patient OR portal OR engagement OR participation OR involvement OR "portal use" OR "portal usage" "patient portal"
5	Return articles dated between:	2000-2020 (exclude citations and patents)
