# Supplementary material for: Patients Managing Their Medical Data in Personal Electronic Health Records: Scoping Review
Source: J Med Internet Res. 2022 Dec 27;24(12):e37783. doi: 10.2196/37783 (PMC9832357; doi:10.2196/37783)
Supplement: Multimedia Appendix 3 [file jmir_v24i12e37783_app3.pdf]

Multimedia Appendix 3

Data Extraction Form

**Study ID (see # Excel):**		
First author:	Data extractor (initials):	Date form completed:

**First check of Eligibility**	
**Report characteristics**	
**A. Type of publication**	**Include**: Practice briefs, fact sheets, white papers, and peer-reviewed publications and conference proceedings.<br>**Exclude**: systematic or scoping review; meta-analysis
	☐ Journal Article (peer-reviewed) ☐ Journal Article (not peer-reviewed) ☐ Conference Proceeding ☐ Practice brief/white paper/opinion letter ☐ Review Paper
	Does the type of publication meet the criteria for inclusion? Yes ☐ No ☐ (**Exclude**) Unclear ☐
**B. Year of publication**	**Include:** 2000 -- 2020
**C. Language of publication**	**English, Dutch**
**Study characteristics**	
**D. Country**	**Specify: _____________________**
**E. Type of study**	**Include any type of intervention**
	**Extra info:**
**F. Duration of study (if applicable)**	**Specify: _____________________**
**G. Aim of the study**	**Specify: _____________________**
**H. Study population and sample size**	**Include:** all (out)patients, clinicians
	☐ Codes **Number of participants:** ________________________<br>**Number of clinicians:**__________________________<br>**Specify (sample size that actively uses portal):** _______________________________________
	**Medical condition/chronic disease patient:** ☐ Codes **Specify: _____________________** **Age group patient** ☐ Codes **SES/Ethnicity/Race** ☐ Codes
	Does the population meet the criteria for inclusion? Yes ☐ No ☐ (**Exclude)** Unclear ☐

**I. PGHD mentioned and duration of measurement**	**Include:** patients who view and/or manage (core) health data into an electronic patient portal<br>**Exclude**: when (core) health data is not being viewed and/or managed by patients themselves in an electronic portal/ thus also exclude symptom reporting/PROMS<br><br>**Duration of measured data management:**<br><br>☐ Measured only once at the time of the study<br>☐ Measured over a time span of ______ years/months
	Does the intervention meet the criteria for inclusion? Yes ☐ No ☐ (**Exclude**) Unclear ☐

**Data extraction (only fill out if report is included)**	
**Type of PGHD/core health data**	
**A. Type of core health data entered in PEHR**	☐ Codes
**Type of personal electronic health record**	
**B. Type of application/system used to record core health data**	☐ Codes<br>**Specify:_______________________________**
**C.1. Accessed via**	☐ Codes
**C.2. Tools for data entry**	☐ Codes
**Patients' Actions with PEHR**	
**C. Patients actions with the PEHR**	☐ Codes
**D. Measured Frequency of PGHD**	**Specify: _______________________________**
**Facilitators**	
**E. For patients**	☐ Codes<br>**Specify: _______________________________**

**F. For care providers**	☐ Codes<br>**Specify: ________________________________**
**G. PEHR-related**	☐ Codes<br>**Specify: ________________________________**
**Barriers**	
H. **Patient related**	☐ Codes<br>**Specify: ________________________________**
**I. Care provider-related**	☐ Codes<br>**Specify: ________________________________**
**J. PEHR-related**	☐ Codes<br>**Specify: ________________________________**
**Clinical Effects: Quality and safety of care**	
K. Safety patient care	☐ Codes<br>**Specify: ________________________________**
L. Decision making processes	☐ Codes<br>**Specify: ________________________________**
M. Patient and care provider satisfaction	☐ Codes<br>**Specify: ________________________________**
N. Cost effectiveness	☐ Codes<br>**Specify: ________________________________**

General comments
